# Therapeutically Engineering Exosomes to Target CD206+ M2 Macrophages to Prevent the Development of Primary Tumors and Distal Metastases in Breast Cancers

**DOI:** 10.3390/cancers18101619

**Published:** 2026-05-16

**Authors:** Mahrima Parvin, Ahmet Alptekin, Sawaiz Kashif, Fowzia A. Selina, Mst Anika Bushra, Mohammad Syam, Mohammad H. Rashid, Alicia Arnold, Yutao Liu, Santhakumar Manicassamy, Hasan Korkaya, Ali S. Arbab

**Affiliations:** 1 Georgia Cancer Center, Department of Biochemistry and Molecular Biology, Augusta University, Augusta, GA 30921, USAfselina@augusta.edu (F.A.S.);; 2 Department of Medicine, Carle Illinois College of Medicine, Urbana, IL 61801, USA; 3 Department of Surgery, Medical College of Georgia, Augusta University, Augusta, GA 30912, USA; 4 Department of Cellular Biology and Anatomy, Augusta University, Augusta, GA 30912, USA; 5 Karmanos Cancer Institute, Wayne State University, Detroit, MI 48202, USA

**Keywords:** breast cancer, exosomes, tumor associated macrophages, tumor microenvironment, antibody-dependent cellular cytotoxicity

## Abstract

**Background/objective**: Approximately 90% of breast cancer-related deaths result from recurrence and metastasis. Emerging evidence indicates that tumor recurrence, invasion, and metastatic spread are strongly influenced by both the tumor microenvironment (TME) and the metastatic niche. M2 macrophages promote immune suppression, inhibit inflammation, and facilitate epithelial-to-mesenchymal transition, invasion, neovascularization, and tumor progression. These phenomena are particularly pronounced in triple-negative breast cancer (TNBC). The objectives of this study were to develop engineered exosomes to selectively deplete CD206+ M2 macrophages from the TME to delay the growth of primary tumors and distal metastasis and enhance overall survival. **Methods**: Engineered exosomes were developed using our invented platform to selectively target and deplete alternatively activated CD206+ M2 macrophages in primary and metastatic TMEs via antibody-dependent cell-mediated cytotoxicity (ADCC). The engineered exosomes were characterized for size, zeta potential, and successful incorporation of targeting peptides and proteins. Whole-body and tumor-specific biodistribution were assessed. In vitro and in vivo experiments were conducted to evaluate targeting specificity. Toxicity and immunogenicity were examined in immunocompetent animal models. Two treatment paradigms were employed. **Results**: Engineered exosomes containing M2 macrophage-targeting peptides and Fc-mIgG2b were successfully made, and no significant size difference was observed between the engineered and control exosomes. Both in vitro and in vivo studies confirmed the specificity of the engineered exosomes. Biodistribution studies showed no significant uptake or retention by the resident macrophages in the lung and liver. No significant immune activation, based on cytokine profiling, or organ-specific toxicity was observed in immunocompetent models. Flow cytometry studies using splenocytes showed significant depletion of M2 macrophages following treatments with engineered exosomes; however, no effect on the distribution of T cells was observed. M2-targeting engineered exosomes significantly delayed the post-resection recurrence and metastasis of tumors, and improved animal survival. **Conclusions**: These findings support the potential of precision exosome-based strategies for enhancing therapeutic outcomes in breast cancer.

## Simple Summary

Approximately 90% of breast cancer-related deaths result from recurrence and metastasis. Emerging evidence indicates that tumor recurrence, invasion, and metastatic spread are strongly influenced by both the tumor microenvironment (TME) and the metastatic niche. Recurrent and metastatic breast cancers show a higher number of immunosuppressive CD206+ M2 macrophages in the TME. The aim of this study was to develop engineered exosomes carrying CD206+ M2 macrophage-targeting peptides and therapeutic payloads (mFc-IgG2b) to deplete CD206+ M2 macrophages through antibody-dependent cell-mediated cytotoxicity to delay the growth of primary and metastatic breast cancers. We successfully developed engineered exosomes that depleted M2 macrophages from the breast cancer TME and subsequently delayed the post-resection recurrence and metastasis of breast cancers.

## 1. Introduction

Approximately 90% of breast cancer-related deaths are attributable to recurrence and metastasis. Emerging evidence indicates that tumor recurrence, invasion, and metastatic spread are strongly influenced by both the tumor microenvironment (TME) and the metastatic niche [1]. The TME consists of a dynamic and complex network of cancer cells, stromal cells, immune cells (including myeloid cells, T cells, and B cells), and endothelial cells. These components interact through the extracellular matrix, soluble mediators such as cytokines and chemokines, and environmental factors, including hypoxia [2,3,4,5]. Within this milieu, interactions among T cells, B cells, natural killer (NK) cells, macrophages, granulocytes (including neutrophils), and dendritic cells can either promote or inhibit tumor progression [3,5,6,7]. Macrophages exhibit remarkable plasticity and undergo functional reprogramming in response to environmental cues, broadly categorized into two polarization states: M1 (classically activated) and M2 (alternatively activated and immunosuppressive) [8,9]. M1 macrophages typically express elevated levels of major histocompatibility complex class II (MHC II), CD68, and the co-stimulatory molecules CD80 and CD86, but lack CD206 expression. In contrast, M2 macrophages express elevated levels of MHC II, CD163, CD206 (also known as MR or MRC1), and in mice arginase-1 (Arg-1), among other markers. Functionally, M2 macrophages secrete elevated levels of IL-10 and transforming growth factor-β (TGF-β), along with low levels of IL-12 and IL-23, reflecting a type-2 cytokine profile. They also produce chemokines such as CCL17, CCL22, and CCL24, which together promote recruitment of regulatory T cells (Tregs), Th2 cells, eosinophils, and basophils, thereby establishing a highly immunosuppressive TME [10,11]. Clinical studies have demonstrated that tumors with a higher number of CD206+ macrophages are associated with poor prognosis, including reduced overall and disease-free survival [12,13,14]. CD206 is a 175 kDa type I transmembrane protein expressed on alternatively activated M2 macrophages and certain tissue-resident macrophages, particularly in the lungs, spleen, and liver [15]. Based on these observations, we hypothesize that selective targeting and depletion of CD206+ M2 macrophages using a therapeutic strategy could reprogram the immunosuppressive TME, inhibit tumor growth and metastatic progression, and improve survival in breast cancer.

Exosomes are a subtype of extracellular vesicles (EVs), measuring 30–150 nm in diameter, that are secreted by most cell types into the extracellular space. They modulate the microenvironment through cell-to-cell communication by fusing with the plasma membrane, undergoing endocytosis, and releasing their molecular cargo into recipient cells [16,17,18,19,20,21]. Regardless of their cellular origin, exosomes share common structural and molecular features, including tetraspanins (CD9, CD63, and CD81), heat shock proteins (Hsp60, Hsp70, and Hsp90), biogenesis-associated proteins (Alix and TSG101), membrane transport and fusion proteins (GTPases, annexins, and Rab proteins), nucleic acids (mRNAs, miRNAs, long non-coding RNAs, and DNA), and lipids such as cholesterol and ceramide [17,22,23]. Due to their intrinsic biocompatibility, low toxicity and immunogenicity, the ability to cross biological barriers including the blood–brain barrier (BBB), stability in circulation, and preferential accumulation at pathological sites, exosomes have emerged as promising therapeutic delivery vehicles [24,25,26,27,28,29,30]. Consequently, researchers have developed engineered exosomes to display biologically active proteins on their surface or encapsulate therapeutic agents for targeted delivery [26,31,32,33,34]. Recently, our laboratory has achieved several advances in exosome technology: (1) development of a proprietary platform (US Patent Application 17/083,124 [35]) for generating engineered exosomes that express specific cell-targeting peptides for in vivo detection; (2) application of these engineered exosomes as therapeutic probes to selectively deplete target cells; (3) optimization of scalable methods for rapid production of large quantities of uniform-sized exosomes from diverse cell sources; and (4) demonstration of differential biodistribution of exosomes in tumor-bearing models using clinically relevant single-photon emission computed tomography (SPECT) imaging [36,37]. We hypothesize that selectively targeting CD206+ M2 macrophages within the breast cancer TME using engineered exosomes functionalized with an M2-specific targeting moiety and the Fc portion of mouse IgG2b (Fc-mIgG2b) to induce antibody-dependent cell-mediated cytotoxicity (ADCC) will reprogram the immunosuppressive microenvironment, inhibit tumor growth and metastasis, and improve survival outcomes.

In recent years, investigators have identified a peptide sequence, CSPGAK (6 amino acid, aa), that targets both mouse and human cells and its cyclic form CSPGAKVRC (9aa), that binds specifically to CD206+ M2 macrophages in tumors and sentinel lymph nodes in different tumor models [38,39]. The primary objective of this study was to evaluate the efficacy of non-tumorigenic HEK293 cell-derived engineered exosomes to selectively target and deplete the alternatively activated CD206+ M2 macrophages within primary and metastatic TMEs in vivo via ADCC. We further aimed to determine whether modulation of the immunosuppressive TME using this strategy could reduce tumor burden and enhance survival in animal models.

## 2. Materials and Methods

### 2.1. Ethics Statement

This study was conducted in compliance with the Animal Research: Reporting of the In Vivo Experiments (ARRIVE) Essential 10 guidelines, ensuring reporting of all the essential items for reproducibility and study design. All the experiments were performed according to the National Institutes of Health (NIH) guidelines and regulations. The Institutional Animal Care and Use Committee (IACUC) of Augusta University (protocol #2014–0625) approved all the experimental procedures.

All the animals (6–8 weeks old, Balb/c and C57BL/6 mice from Charles River, Frederick, MD, USA) were kept under regular barrier conditions at room temperature with exposure to light for 12 h and dark for 12 h. Food and water were offered ad libitum. The humane endpoint of the survival studies was the fulfillment of the criteria for euthanasia at the end of the survival studies (survival) according to the IACUC-approved protocol. All efforts were made to ameliorate the suffering of the animals. CO_2_ (displacement rate of 30–70% of the chamber volume with CO_2_ per minute), with a secondary method (bilateral thoracotomy or collection of major organs), was used to euthanize animals for tissue collection. Death was confirmed by the established criteria of lack of breathing, lack of corneal reflex, and lack of response to a firm toe pinch. All the mice were randomized. Studies were conducted in multiple batches, and each batch had animals for experimental and corresponding control groups. Personnel blinded to the various groups analyzed the data. The animals were of the same age and weight and had similar baseline behavior.

### 2.2. Cell Lines, Peptides, and Proteins

Syngeneic, aggressive triple-negative breast cancer (TNBC) mouse cell lines 4T1 (for Balb/c mice, ATCC, Manassas, VA, USA) and AT3 (for C57BL/6, Millipore, Burlington, MA, USA) were obtained and maintained in the laboratory in Dulbecco’s Modified Eagle Medium (DMEM) (Corning, Corning, NY, USA) supplemented with 10% fetal bovine serum (FBS) (Nalgene-GIBCO, Rochester, NY, USA), sodium pyruvate (GIBCO, Grand Island, NY, USA), non-essential amino acids (GIBCO), sodium glutamate (GIBCO), and 100 U/mL penicillin and streptomycin (GIBCO). The cells were harvested for implantation at 75–80% confluence. Approximately 2.5 × 10^4^ cells suspended in 20 µL of Matrigel (Corning) were orthotopically implanted into the lower right mammary fat pad of mice (see below) under anesthesia.

HEK293 cells (ATCC) were used to produce engineered exosomes. These cells were transduced with lentiviral vectors encoding CD206+ M2 macrophage-targeting peptides and Fc-mIgG2b, enabling surface expression of these constructs on the generated exosomes. Primary mouse bone marrow-derived macrophages (MBMDMs) were isolated from tumor-bearing mice, cultured in vitro, and polarized toward an M2 phenotype using GM-CSF (40 ng/mL), IL-13 (20 ng/mL), and IL-4 (20 ng/mL) (all from Prospec, East Brunswick, NJ, USA). Commercial mouse macrophage cell lines Raw 264.7 (ATCC) and J774A.1 (ATCC) were used for in vitro experiments and similarly polarized to the M2 phenotype with IL-13 and IL-4. The cells were stimulated with the growth factor and cytokine for 3–4 days.

To evaluate the specificity of the precision peptide-carrying engineered exosomes, targeting peptides (6-amino-acid and 9-amino-acid sequences for blocking studies) and a fusion protein consisting of the 9-amino-acid targeting peptide linked to Fc-mIgG2b were synthesized by a commercial vendor (GeneScript, Piscataway, NJ, USA).

### 2.3. Experimental Animal Models

For this study, syngeneic metastatic TNBC models were established in immunocompetent female Balb/c (Charles River, Frederick, MD, USA, age 6–8 weeks) mice using 4T1 cells (aggressive TNBC) and in female C57BL/6 mice (Charles River, age 6–8 weeks) using AT3 cells (slow-growing TNBC). The mice were randomized before treatment allocation. The experiments were performed in multiple batches, with each batch including both experimental and corresponding control groups. Data analysis was conducted by personnel blinded to group assignments. Treatment was initiated on day 8 after tumor implantation, when most tumors measured approximately 3 mm in diameter.

To generate a resection model for evaluating tumor recurrence and metastasis, primary tumors were surgically removed under anesthesia on day 11 post-implantation, following the protocol described in our previously published report [40]. We used a total of 54 Balb/c and 57 C57BL/6 female mice.

### 2.4. Biogenesis, Characterization, and Determination of In Vitro Specificity of Engineered Exosomes

Supplementary Figure S1 illustrates the vector design and a schematic representation of ADCC mediated by the engineered exosomes. Plasmids encoding CD206+ M2 macrophage-targeting peptides (6-amino-acid or 9-amino-acid sequences) and Fc-mIgG2b were generated by a commercial provider (VectorBuilder, Chicago, IL, USA). Lentiviral particles carrying these constructs were produced in-house using HEK293TN cells and a standard packaging system (Addgene, Watertown, MA, USA).

The HEK293 cells were transduced with the lentiviral vectors and selected using puromycin. Successful transduction was confirmed by fluorescence microscopy based on mCherry expression. Stably transduced (mCherry^+^) cells were then cultured in exosome-depleted media, and conditioned supernatants were collected at 48 and 72 h following media replacement. Typically, 6 × 10^6^ cells were seeded in a T75 flask, yielding approximately 2 × 10^10^ exosomes over 3 days.

Exosomes were isolated and concentrated from the collected supernatants using our previously published and optimized protocols [36,37]. Purified exosomes were aliquoted and stored at −20 °C until further use. Supplementary Figure S2 shows the integrity of frozen engineered exosomes after 18 to 24 months. Genomic DNA extracted from the transduced (mCherry^+^) cells was analyzed by polymerase chain reaction (PCR) to confirm the presence of inserted sequences encoding the M2 macrophage-targeting peptides and Fc-mIgG2b. Primers were obtained from Integrated DNA Technologies (IDT, Coralville, IA, USA). The primer sequences were as follows:

CD206 9aa peptide (CSPGAKVRC)

Forward: 5′-TGCTCTCCGGGGGCGAAA-3′.

Reverse: 5′-CAGTCCTGGTGCTGGATGGG-3′.

Fc-mIgG2b

Forward: 5′-GGGCCCATTTCAACAATCAACC-3′.

Reverse: 5′-CTGATGTCTCCAGGGTTGAAGCCC-3′.

Each batch of engineered exosomes was characterized by nanoparticle-tracking analysis (NTA) to determine particle size distribution and zeta potential. Western blot (WB) analysis was performed to confirm the presence of exosomal markers (e.g., CD9). Immuno-transmission electron microscopy (TEM, JEOL JEM-1400 Flash Transmission Electron Microscope with Gatan 1095 One View Camera (JEOL USA, Inc, Peabody, MA, USA) was used to assess exosome morphology, size, and surface expression of the inserted Fc-mIgG2b. For immuno-TEM, a specific antibody against Fc-mIgG2b (Jackson ImmunoResearch Laboratories, Inc., West Grove, PA, USA) was utilized.

Mouse embryonic fibroblasts (MEFs, negative control) and MBMDMs were used to assess targeting specificity. After plating and polarization of MBMDMs to the M2 macrophage phenotype, DiI-labeled engineered exosomes were added and incubated for 1–2 h. For blocking experiments, polarized cells were pre-incubated with CD206+ M2 macrophage-targeting peptides before the addition of the engineered exosomes. Following incubation, the cells were washed with PBS, fixed with paraformaldehyde, and counterstained with DAPI. Fluorescence images were captured from randomly selected fields and quantified using the ImageJ software v1.54s (NIH, Bethesda, MD, USA) (color threshold method).

To evaluate whether the engineered exosomes remained on the cell surface, murine macrophage J774A.1 cells were polarized to the CD206+ M2 phenotype and incubated with DiI-labeled engineered exosomes. After washing, the cells were cultured in exosome-free media and collected at 0.5, 1, 3, 6, and 24 h. The cells were fixed, counterstained with DAPI, and examined by fluorescence microscopy to determine the localization of DiI-labeled exosomes on the cell surface.

### 2.5. Biodistribution of Control and Engineered Exosomes

CD206 is a 175 kDa type I transmembrane protein expressed by alternatively activated M2 macrophages and tissue-resident macrophages, particularly in the lungs, spleen, and liver [15]. Given this expression pattern, it was essential to determine whether CD206-targeting engineered exosomes preferentially accumulate in organs enriched with resident macrophages, such as the lungs and liver.

Control exosomes (derived from HEK293 cells) and engineered exosomes were radiolabeled with iodine-131 (I-131) according to our previously published protocol [36]. A total of 10 female Balb/c mice were used. The radiolabeled control or engineered exosomes were administered intravenously, and the animals underwent SPECT imaging at 5 min, 3 h, and 24 h post-injection. Whole-body SPECT images were acquired using a dedicated four-head NanoScan high-sensitivity microSPECT/CT 4R system (Mediso, Boston, MA, USA) equipped with high-resolution multi-pinhole collimators (100 pinholes total). The microSPECT system operates across an energy range of 20–600 keV and provides a spatial resolution of 275 µm. Imaging was performed using 60 projection views at 30–60 s per projection with a medium field of view. Attenuation correction was applied using concurrently acquired CT images, followed by image reconstruction with low-iteration, low-filtered back projection. The animals were maintained under anesthesia throughout the imaging procedure. Body temperature was maintained at 37 °C, and respiration was continuously monitored. After image reconstruction, radioactivity in the whole body, lungs, heart, and liver was quantified using the ImageJ software (NIH). Organ-specific activity (lungs, heart, and liver) was normalized to total whole-body activity and expressed as a percentage of whole-body activity. The biodistribution profiles of the control and engineered exosomes in the lungs, liver, and heart were then compared.

### 2.6. In Vivo Specificity by SPECT Imaging

The in vivo specificity of CD206+ M2 macrophage-targeting peptides and engineered exosomes has been previously reported [37]. In the present study, we further evaluated the specificity of engineered exosomes and a CD206-targeting fusion protein and compared them with control exosomes (HEK293-derived) and anti-CD206 antibodies.

A total of 10 female Balb/c mice bearing metastatic breast tumors were included (*n* = 3 engineered exosomes, *n* = 3 fusion protein, *n* = 2 HEK293 control exosomes, *n* = 2 anti-CD206 antibody). The metastatic breast cancer model was established in Balb/c mice, and SPECT imaging was performed 5 weeks after tumor implantation to assess whole-body biodistribution and distribution to primary tumors and metastatic lesions. The control and engineered exosomes, fusion protein, and anti-CD206 antibody were radiolabeled with I-131 according to our previously published protocols [36,41]. The radiolabeled agents were administered intravenously, and SPECT/CT imaging was obtained 3 h post-injection, as described in our reported studies [36,37].

### 2.7. In Vivo Depletion of CD206+ M2 Macrophages and CD11b^+^ Myeloid Cells

The in vivo specificity and dose-escalation studies of CD206+ M2 macrophage-targeting peptides and engineered exosomes were previously reported [37]. In the present study, we investigated the specificity of engineered exosomes and fusion proteins and compared them with that of HEK293 exosomes and anti-CD206 antibodies for depleting M2 macrophages and T cells. Three doses (3 days/week) of control exosomes (HEK exo), engineered exosomes (CD206 exo, 3 × 10^9^ exosomes/dose), fusion protein (50 µg/day), and anti-mCD206 antibody (50 µg/day) were administered in the respective group of animals (Balb/c, *n* = 3 per group). Spleens were collected 24 h after the last dose, and a single-cell suspension was made. Different immune cell populations, including CD206+ M2 macrophages and CD11b^+^ myeloid cells, were quantified by flow cytometry.

### 2.8. Immunogenicity and Toxicity of HEK293 Cell-Derived Engineered Exosomes

To evaluate potential immunogenic and inflammatory responses following systemic administration of the engineered exosomes, non-tumor-bearing female Balb/c (*n* = 3) and C57BL/6 (*n* = 3) mice received six intravenous doses of either PBS (vehicle control) or engineered exosomes over a period of 6 weeks (one dose per week). Twenty-four hours after the final dose, the animals were euthanized, and blood plasma was collected for membrane array analysis to quantify pro-inflammatory, immunogenic, and immunosuppressive cytokines and growth factors.

In a separate long-term effects study, groups of mice (Balb/c and C57BL/6, *n* = 3 per group) were euthanized 6 months after receiving six doses of the engineered exosomes. Blood plasma was collected to assess the presence of mouse anti-human antibodies (MAHA). Major organs, including the liver, spleen, lungs, kidneys, and heart, were harvested for histopathological evaluation using hematoxylin and eosin (H&E) staining to assess potential cellular or tissue-level toxicities. The rationale behind this investigation was to investigate whether HEK293 cell-derived exosomes would initiate antibody formation by the host (mouse) and affect major organs through antibody-mediated toxicity.

### 2.9. In Vivo Validation of NK Cell-Mediated ADCC

In our previous study, we demonstrated that splenocytes containing NK cells contribute to the depletion of polarized M2 macrophages in vitro [37]. In the present study, we investigated whether in vivo depletion of CD206+ M2 macrophages by engineered exosomes was dependent on NK cell-mediated ADCC. An orthotopic AT3 breast cancer model was established in female C57BL/6 mice. Beginning on day 8 after tumor implantation, randomly assigned animals received four doses of one of the following: vehicle (PBS; *n* = 3), isotype IgG (*n* = 3), anti-Gr1 antibody (*n* = 3), or anti-NK antibody (PK136; *n* = 3). Subsequently, all the groups were treated with four doses of engineered exosomes over a period of 2 weeks to deplete CD206+ M2 macrophages.

Tumor volumes and optical images were obtained on days 8 (baseline), 15, and 22 after tumor implantation. The rationale of the study was that if depletion of CD206+ M2 macrophages is mediated through NK cell-dependent ADCC, then the NK cell-depleted mice (anti-NK-treated group) would fail to reduce M2 macrophage accumulation in the TME following administration of the engineered exosomes, and tumor growth inhibition would not occur. In contrast, vehicle-, isotype IgG-, and anti-Gr1-treated animals without NK cell depletion would retain NK cell function, allowing engineered exosomes to reduce M2 macrophage levels in the TME and consequently suppress tumor growth.

### 2.10. Investigating the Changes in the TME Milieu and Cytokine Levels

An orthotopic syngeneic AT3 breast cancer model was established in C57BL/6 female mice. Following tumor implantation, the animals were treated with the engineered exosomes (3 × 10^9^ exosomes per dose, twice weekly) administered either intratumorally (local delivery) or intravenously (IV). A control group received targeting exosomes administered IV that carried the targeting peptide alone (without mFc-IgG2b). The treatments continued for 2 or 4 weeks. At the respective endpoints, blood plasma, lungs (metastatic site), and primary tumors were harvested, and total protein was extracted. A custom-designed membrane array was used to quantify pro-inflammatory and immunosuppressive cytokines, chemoattractants, and growth factors, as previously described [36,42]. We also made single-cell suspensions from the collected lungs, tumors, and spleens to determine the number of CD206+ M2 macrophages. A total of 15 animals were used.

In a separate experiment, an orthotopic syngeneic 4T1 breast cancer model was generated in Balb/c female mice. The animals were treated with either vehicle (PBS) or engineered exosomes (3 × 10^9^ exosomes per dose, twice weekly for 3 weeks). At the end of the study, single-cell suspensions were prepared from the primary tumor, lungs (potential metastatic site), and spleen. Both surface and intracellular markers were analyzed by flow cytometry to characterize myeloid, T-cell, and dendritic cell populations. Each group included three animals.

### 2.11. Effect of Engineered Exosomes on Tumor Growth, Recurrence, Metastasis, and Survival

Previous preclinical studies have demonstrated an increase in immunosuppressive cell populations within primary and metastatic breast tumors following chemotherapy and radiotherapy [43,44,45,46,47]. Clinical investigations have similarly reported elevated levels of immunosuppressive cells, including CD206+ M2 macrophages, in primary, metastatic, and therapy-resistant breast cancers [43,48,49]. Based on these findings, we hypothesized that depletion of CD206+ M2 macrophages within the TME would delay primary tumor growth and improve overall survival. We used both the AT3 and 4T1 tumor models that were tested for all the experiments, and the representative results have been included.

Orthotopic AT3 tumor-bearing C57BL/6 mice were treated with either control or engineered exosomes (3 × 10^9^ exosomes per dose, twice weekly) for 3 weeks, beginning on day 8 post-implantation. Tumor growth and survival were monitored throughout the study. Tumor volume, body weight, and general health status were assessed at least twice weekly until predefined euthanasia criteria were met. Each group consisted of 4 animals.

In a post-surgical model, Balb/c female mice (*n* = 10) were orthotopically implanted in the lower right mammary fat pad with luciferase-expressing 4T1 breast cancer cells. Eleven days after tumor cell implantation, primary tumors were surgically resected. Twenty-four hours after surgery, all the animals underwent bioluminescence imaging (BLI) to assess residual tumor burden. The animals with substantial residual disease were excluded from further analysis. The remaining mice were treated IV with either the HEK293 exosomes or engineered exosomes at a dose of 3 × 10^9^ exosomes per injection. The treatments were initiated 24 h after tumor resection and administered weekly. The animals were monitored by serial BLI for the development of lung metastases or recurrence at the primary tumor site. Supplementary Figure S3 shows the development of recurrence and metastatic foci over time.

To determine the effect of frozen engineered exosomes in a post-surgical model, C57BL/6 female mice (*n* = 16) were orthotopically implanted into the lower right mammary fat pad with luciferase-expressing AT3 breast cancer cells. On day 11 post-implantation, primary tumors were surgically resected. Twenty-four hours after surgery, all the animals underwent BLI to assess residual tumor burden. The animals with substantial residual disease were excluded from further analysis. The remaining mice were treated with either the HEK293 exosomes or 18-month-old (frozen) engineered exosomes at a dose of 3 × 10^9^ exosomes per injection. The treatments were initiated 24 h after tumor resection and administered weekly, and survival of the animals was noted until 75 days after implantation of the tumor (end point of the study).

### 2.12. Statistical Analysis

All the data were expressed in mean ± standard error of mean (SEM) unless otherwise stated. The effects of the treatments (in vitro and in vivo) were analyzed using ANOVA across the treatment groups, followed by pairwise comparisons with Tukey’s adjustment. Bonferroni corrections were applied to multiple related parameters. For the analysis of 53 cytokines/chemokines, a non-parametric 4-group comparison with Holm correction was applied. Survival studies were analyzed by the Kaplan–Meier estimate. A *p*-value of <0.05 was considered a significant difference. We used GraphPad Prism 10 for statistical analysis.

## 3. Results

### 3.1. Biogenesis of Engineered Exosomes and Determination of Specificity

**Figure 1:**
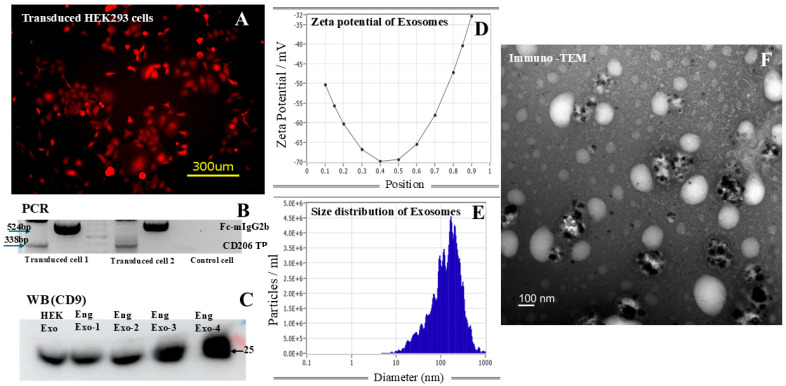
Biogenesis of engineered exosomes and their characteristics: A lentivector containing plasmids that express CD206-targeting peptides and Fc-mIgG2b on the exosomes was used to transduce HEK293 cells. (**A**) mCherry-positive cells are indicative of transduced HEK293 cells. The yellow bar represents 300 µm. (**B**) PCR was performed to determine the expression of the inserted sequences in the DNA of the transduced cells. The transduced HEK293 cells expressed the sequences of both CD206-targeting peptide (TP, 338 bp) and Fc-mIgG2b (524 bp). (**C**) Collected exosomes from different transduced cells showed the classical marker of exosomes (CD9) on Western blotting. CD9 is expressed in the control HEK293 (HEK Exo) and engineered exosomes (Eng Exo). Nanoparticle-tracking analysis showed typical (**D**) zeta potential and (**E**) size distribution of the collected engineered exosomes. (**F**) Immuno-transmission electron microscopy showed the expression of Fc-mIgG2b (black dots) on the surface of the engineered exosomes (white oval-shaped structures). The TEM can also confirm the size of the exosomes. The white bar represents 100 nm. The original PCR and WB gels can be found in Supplementary Materials. PCR = polymerase chain reaction; WB = Western blot; HEK Exo = HEK293 cell-derived control exosome; Eng Exo = engineered exosome.

**Figure 2:**
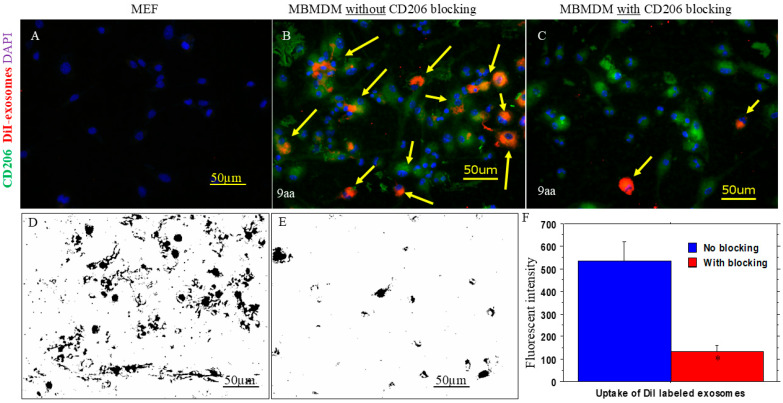
Specificity of engineered exosomes: Mouse embryonic fibroblasts (MEFs) were used as a negative control, and polarized mouse bone marrow-derived macrophages (MBMDMs) were used as a positive control. Uptake of the engineered exosomes (DiI-labeled, red) was investigated with or without blocking peptides. (**A**) MEFs did not show any uptake of the DiI-labeled exosomes. Blue colored areas are cell nucleus (DAPI) (**B**) Polarized MBMDMs (green) showed plenty of engineered exosome (red, yellow arrows) uptake without blocking. (**C**) Polarized MBMDMs (green) showed an exceptionally low level of exosome (red, yellow arrows) uptake with blocking. Yellow arrows shown in (**B**,**C**) indicate DiI-tagged exosomes (red) in the MBMDMs. (**D**,**E**) Color threshold-masked images from without or with blocking of CD206. (**F**) Quantitative analysis shows significantly (* *p* < 0.01) lower uptake following blocking of CD206, indicating the specificity of the engineered exosomes. Student’s *t*-test was applied. Panels (**D**,**E**) represent color threshold-masked images generated by ImageJ to highlight and quantify signal intensity following treatment with CD206-targeting exosomes, either in the absence or presence of CD206 receptor blocking. These masked images were included to visually emphasize differences in signal localization and intensity that may not be readily apparent in the unprocessed images. *n* = 3 per group. MEFs = mouse embryonic fibroblasts; MBMDMs = mouse bone marrow-derived macrophages.

Figure 1 shows the representative images indicating successful generation of engineered exosomes carrying both M2 macrophage-targeting peptides (9aa) and Fc-mIgG2b. Our selection process produced almost 100% mCherry-positive cells. DNA collected from the transduced cells shows the presence of sequences corresponding to the inserted peptides and Fc-mIgG2b. We did not observe any significant size differences between the control and engineered exosomes (146 ± 125 nm vs. 133 ± 72.3 nm). The Western blot study also showed the presence of CD9 markers in the collected exosomes. Immuno-TEM confirmed the size of the exosomes and the presence of Fc-mIgG2b on the surface of the exosomes. We also produced engineered exosomes using M2 macrophage-targeting 6aa peptides and compared them with those of 9aa peptides with respect to specificity using MBMDMs. The in vitro studies using primary MBMDMs polarized to M2 macrophages showed uptake of the engineered exosomes (carrying either 6aa or 9aa peptides), whereas MEFs showed no uptake of exosomes at all (Figure S4). When the polarized macrophages were pre-incubated with 6aa or 9aa blocking peptides before adding the corresponding engineered exosomes, the uptake of the engineered exosomes carrying 9aa peptides showed significantly lower uptake by the cells, indicating better specificity (Figure 2 and Figure S4). Based on the in vitro specificity and previously reported in vivo specificity of the engineered exosomes carrying 9aa peptides, we decided to perform subsequent studies using the HEK293 exosomes and engineered exosomes carrying 9aa peptides only.

**Figure 3:**
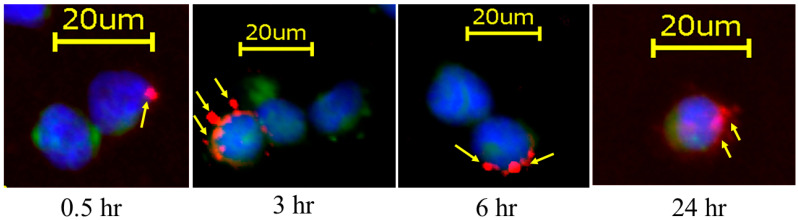
Surface attachment of DiI-labeled engineered exosomes (red) carrying precision peptide and Fc-mIgG2b from 0.5 h to 24 h. Murine macrophages (J774A.1) labeled with CFSE dye (green) were polarized to the M2 macrophage phenotype using IL-13 and IL-4 for 72 h, and then exosomes were added and incubated with the cells. The cells were collected and fixed at different time points and stained with DAPI (blue color). The microscopic fluorescent images showed that the engineered exosomes (red) remained on the surface up to 24 h, indicating that exosomes can bind with NK (natural killer) cells for an extended time. Yellow arrows show the red colored exosomes. *n* = 2 separate culture wells. CFSE = carboxyfluorescein succinimidyl ester, hr = hours.

To determine whether the engineered exosomes remained on the surface of the polarized macrophages for an extended time, DiI-labeled exosomes were added to the culture (glass chamber slides) of the polarized M2 macrophage (J774A.1) cells, and fluorescence microscopic images were taken at 0.5 to 24 h after fixation of the cells. Figure 3 also showed that the engineered exosomes remained on the cell surface for an extended period, indicating that they can function as a bridge to connect the targeted cells with the effector cells for an extended time without being fully phagocytosed or internalized.

**Figure 4:**
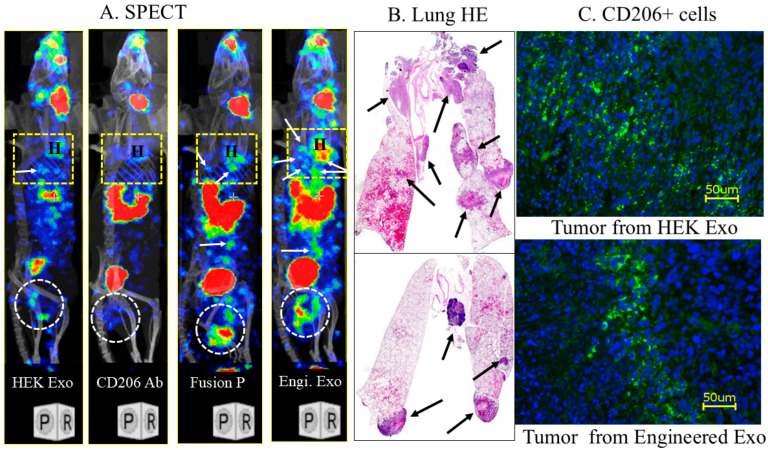
Differential biodistribution of exosomes and protein in 4T1 tumors (Balb/c mice): I-131-labeled control exosomes (HEK293 exo), anti-mouse CD206 antibody (CD206 Ab), fusion protein (fusion P) containing targeting peptide and Fc-mIgG2b, and engineered exosomes (Engi. Exo) carrying targeting peptide were administered IV into 4T1 breast cancer-bearing animals; SPECT images were obtained 3 h after administration. (**A**) SPECT images showed the distribution of the agents in the primary tumors (white circles) and lungs (yellow rectangular areas). White arrows show possible metastatic foci. Fusion protein and engineered exosomes accumulated in the primary and metastatic tumors. We used rainbow color to depict the activity of I-131. Blue is the lowest and red is the highest activity of I-131. (**B**) Randomly selected lung H&E staining showed multiple metastatic foci (black arrows). (**C**) Presence of numerous CD206+ cells is seen in tumors (green cells). *n* = 2–3 animals per group. Ab = antibody, Engi = engineered, Exo = exosomes, HE = Hematoxylin and Eosin, P = protein, SPECT = single-photon emission computed tomography, H = heart.

Figure 4 shows that the engineered exosomes detected both primary tumors (white circles) and multiple metastatic foci in the lungs (white arrows) and other parts of the body (white arrows). The fusion protein showed comparable results but fewer foci in the lungs, even though all the animals showed multiple lung metastases. On the other hand, the anti-CD206 antibody failed to detect primary tumors or lung metastases. The control (HEK293) exosomes showed little uptake in the primary tumor and a few focal activities in the lungs. The randomly selected animals (from the engineered and control exosomes administered) showed multiple lung metastases at 5 weeks following orthotopic tumor implantation in the breast. The primary tumor showed numerous CD206+ (green) cells that received the control or engineered exosomes.

### 3.2. Engineered Exosomes Did Not Show Increased Uptake to the Lungs and Liver

**Figure 5:**
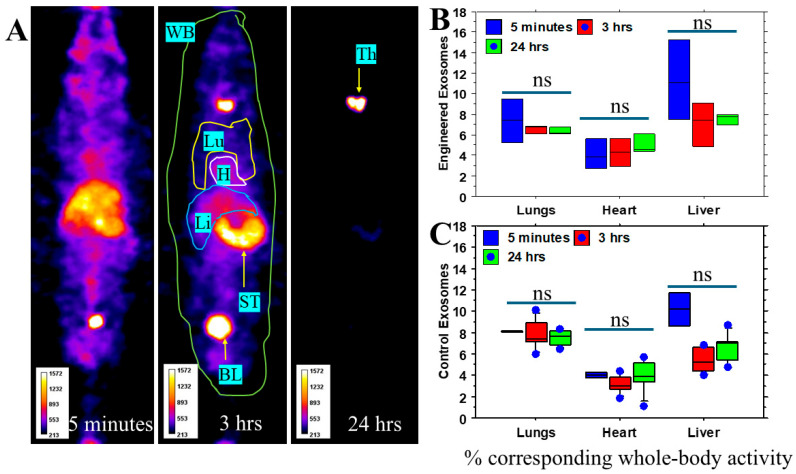
Whole-body and organ-specific biodistribution of administered HEK and engineered exosomes in Balb/c mice: I-131-tagged control and engineered exosomes were administered IV in separate groups of animals. Whole-body (WB) single-photon emission computed tomography (SPECT) images were obtained in 5 min, 3 h, and 24 h. WB and organ-specific (Lu- = lung, H = heart, Li = Liver) activities were determined and expressed as percent WB activity. (**A**) Representative SPECT images showing the distribution of the administered exosomes. (**B**,**C**) Semiquantitative analysis showed similar biodistribution of the I-131-tagged control and engineered exosomes in the heart, lungs and liver of mice at 5 min, 3 and 24 h following administration and no evidence of significant retention of the engineered exosomes was observed in those organs compared to the control exosomes. Activities in the stomach (ST), thyroid (Th), and bladder (BL) are due to free I-131. *n* = 5 per group. One-way ANOVA was applied to determine significance. No significant difference (ns) was observed. BL = bladder, H = heart, hrs = hours, Lu =lungs, Li = liver, ns = not significant, ST = stomach, Th = thyroid, WB = whole body.

We determined the whole-body biodistribution of the I-131-tagged control and engineered exosomes using SPECT scanning. Semiquantitative analysis showed no significant differences between the control and engineered exosomes’ distribution in the lungs and liver from 5 min to 24 h. Activity in the heart also showed no significant differences. Most of the activity from the lungs and livers cleared within 24 h. Figure 5 shows representative images from the engineered exosomes administered to the animals and analysis of the percentage of whole-body activity of the lungs, liver, and heart.

### 3.3. Engineered Exosomes and Fusion Protein (Bispecific Protein) Depleted M2 Macrophages but Not T Cells

**Figure 6:**
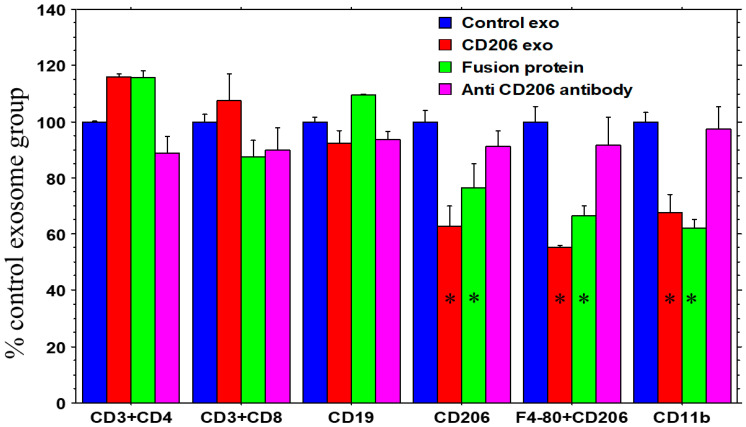
The effect of engineered exosomes (CD206 exo) vs fusion protein vs antibody (in Balb/c mice): Groups of animals were treated separately with control or engineered exosomes (CD206-exo), fusion protein, or anti-CD206 antibody. The animals were euthanized at the end of the treatments, and spleens were collected. Single-cell suspensions were made for flow cytometry using myeloid and T-cell-specific multi-color flow panels. Flow cytometric analysis of splenic cells (collected from the treated animals) showed significantly higher depletion of CD11b+ or CD206+ cells by CD206 exosomes and fusion protein compared to the control exosomes and the antibody-treated group only. There was no significant change in T-cell and B-cell populations. Quantitative data are expressed as mean ± SEM. One-way ANOVA was applied to determine significant difference. * *p* < 0.05 compared to all the groups. *n* = 3 per group. Exo = exosomes.

The engineered exosomes and fusion protein (bispecific protein) depleted CD11b+ or CD206+ macrophages significantly compared to the control exosome- and antibody-treated animals (Figure 6). It is worth noting that the decrease in CD11b+ cells was related to the depletion of CD206+ macrophages, as macrophages are also CD11b+. However, there was no alteration in T cells following the administration of the engineered exosomes or fusion protein. Additionally, the engineered exosomes did not deplete CD16+ or NKp46+ NK cells, and F4/80+/CD80+ M1 macrophages. Flow cytometric strategies are shown in Figures S5 and S6.

### 3.4. Immunogenic or Inflammatory Reactions Were Not Observed Following Administration of HEK293 Cell-Derived Engineered Exosomes

**Figure 7:**
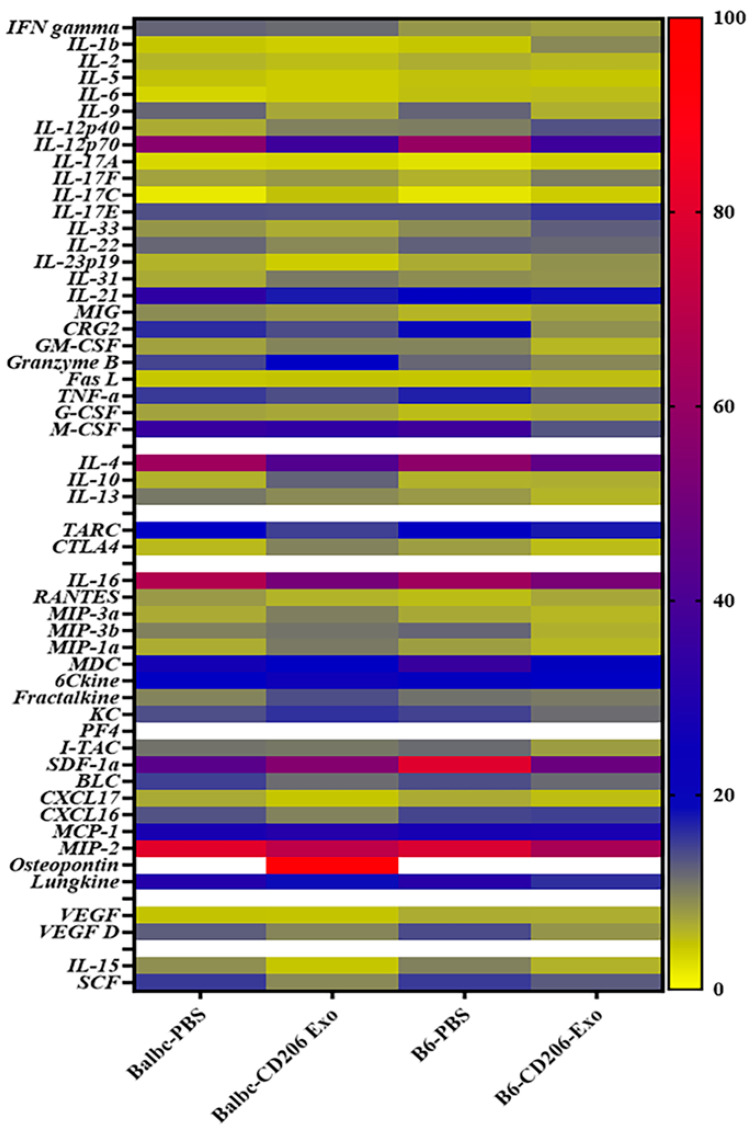
Heat maps of cytokine and chemokine profiles: Both Balb/c and C57BL/6 (B6) animals were treated with 6 weekly doses of either PBS or engineered exosomes (CD206 exo). A total of 24 h after the last dose, the animals were euthanized, and plasma was subjected to membrane array (53 targets) analysis to determine inflammatory, immunogenic, immunosuppressive, invasive, and growth factors. Non-parametric 4-group comparison with Holm correction was applied for the analysis of 53 cytokines/chemokines. No significant differences were found among the groups. *n* = 3 per group. B6 = C57BL/6, Exo = exosomes, PBS = phosphate-buffered saline.

Our studies did not detect mouse anti-human antibody (MAHA) for over 6 months in the immunocompetent animals after IV injections of exosomes (six doses) derived from HEK293 cells. We also found no changes in the expression of pro-inflammatory, immunogenic, and immunosuppressive cytokines, as well as chemoattractants in plasma compared to that of the control animals (PBS-treated) in different immunocompetent animal models (Figure 7).

CD206+ M2 macrophages are present in limited numbers in the lungs, spleen, and liver tissues as resident macrophages [15]. We did not observe any changes at the cellular level in major organs on H&E staining (Figure S7) in both the B6 and Balb/c immunocompetent animal models.

### 3.5. NK Cells Participated in ADCC to Deplete Targeted M2 Macrophages

**Figure 8:**
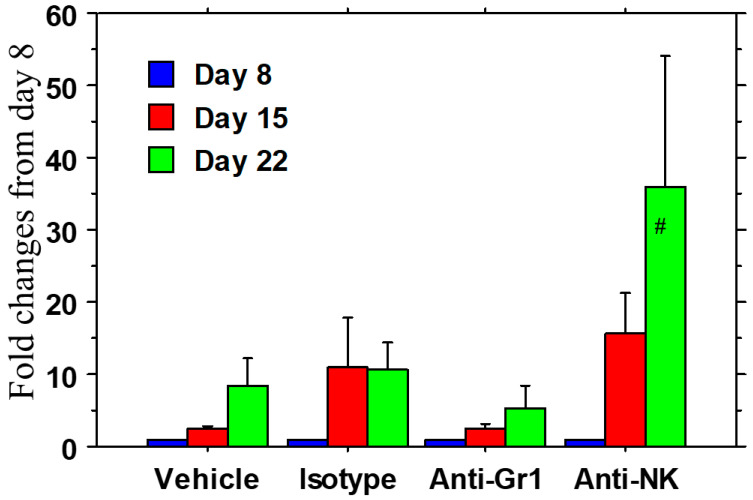
Involvement of NK cells (AT3 tumors in C57BL/6 mice): Tumor volume and optical images were obtained on days 8 (baseline), 15, and 22 following specific cell depletion and engineered exosome therapies. The rationale behind the investigation was that in the absence of NK cells, tumor growth would not decrease even after administration of engineered exosomes. There was a significant (20–40-fold) increase (# = *p* < 0.05) in bioluminescent imaging (BLI) photon intensity in tumors that were treated with the anti-NK antibodies. However, there were no significant changes in photon intensity compared to that of the vehicle or isotype IgG when Gr1+ cells were depleted. Tumor volume measured by calipers also showed comparable results. One-way ANOVA was applied. *n* = 3–4 per group. NK = natural killer cell.

There was a significant (20–40-fold) increase (*p* < 0.05) in tumor volume (BLI photon intensity) in the animals that were treated with the anti-NK antibodies, indicating that NK cell depletion prevented the killing of M2 macrophages through NK-cell-mediated ADCC. However, there were no changes in tumor volume when Gr1+ cells were depleted (Figure 8). It is worth noting that the tumor volume measured by calipers also yielded comparable results.

### 3.6. Differential Changes in the TME Milieu and Cytokine Levels

**Figure 9:**
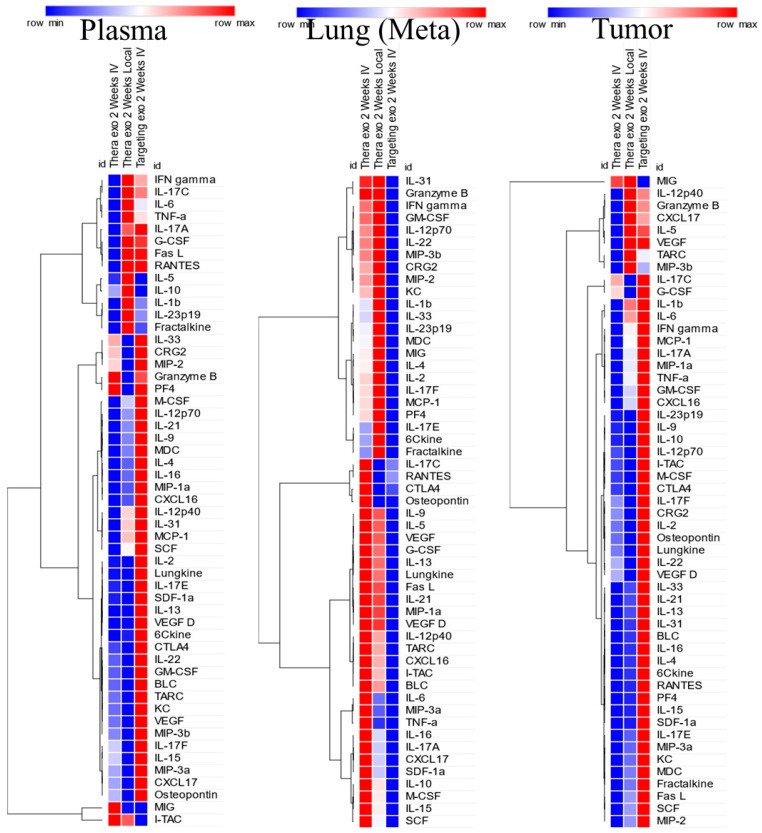
Heat maps of cytokine and chemokine profiles following 2 weeks of treatment (AT3 tumors in C57BL/6 mice): Tumor-bearing animals were treated with the engineered exosomes (either therapeutic (carrying both CD206-targeting peptides and Fc-mIgG2b) or targeting (carrying only CD206-targeting peptides)). The therapeutic exosomes were administered either IV or intratumorally. The plasma and tissues (extracted proteins) from the primary tumors and metastatic sites were subjected to membrane array analysis of 53 targets. The results shown are from pooled samples. The values were normalized to the corresponding targeting exosomes. *n* = 2–3 per group. Increased levels of cytokines and chemokines were observed in the lungs. Targeting Exo = targeting exosomes, Thera-Exo = therapeutic exosomes, Meta = metastasis. Heat maps and analysis were done using Morpheus (https://software.broadinstitute.org/morpheus, accessed on 22 January 2026).

**Figure 10:**
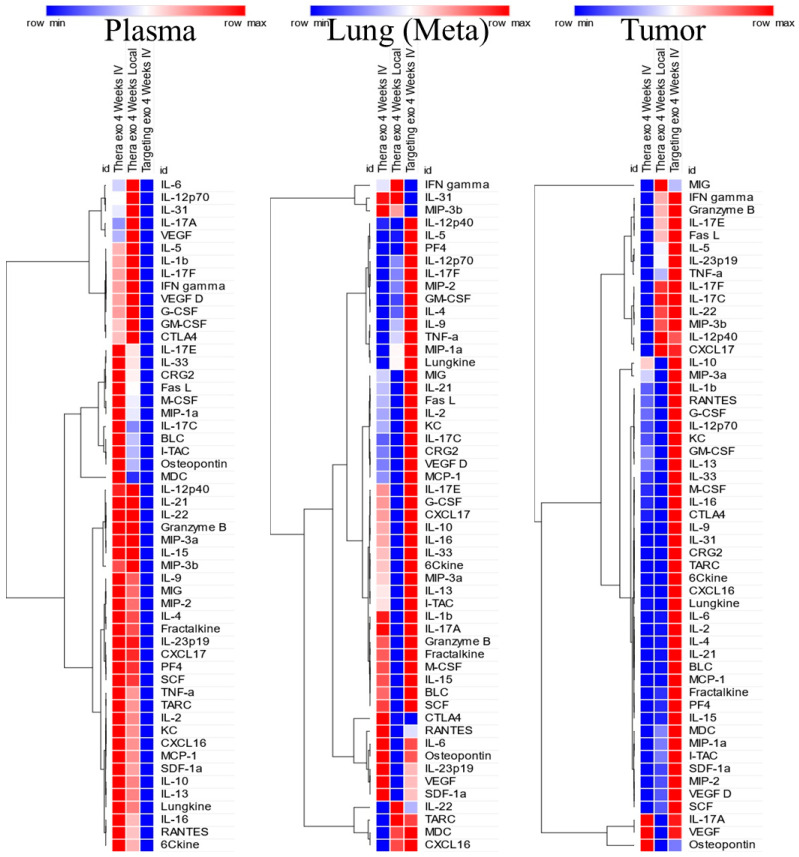
Heat maps of cytokine and chemokine profiles following 4 weeks of treatment (AT3 tumors in C57BL/6 mice). Tumor-bearing animals were treated with engineered exosomes (either therapeutic (carrying both CD206-targeting peptides and Fc-mIgG2b) or targeting (carrying only CD206-targeting peptides)). Therapeutic exosomes were administered either IV or intratumorally. Plasma and extracted protein from the primary tumors and metastatic sites were subjected to membrane array analysis of 53 targets. The results shown are from pooled samples. The values were normalized to the corresponding targeting exosomes. Increased levels of cytokines and chemokines were observed in the plasma. *n* = 2–3 per group. Targeting Exo = targeting engineered exosomes, Thera-Exo = therapeutic engineered exosomes, Meta = metastasis. Heat maps and analysis were done using Morpheus (https://software.broadinstitute.org/morpheus).

The engineered exosomes administered locally or intravenously showed increased (>2-fold compared to the control targeting exosomes) levels of inflammatory cytokines, chemokines, and growth factors at metastatic sites (lungs) following 2 weeks of treatment, which were not seen in the plasma or primary tumors (Figure 9). However, following 4 weeks of therapy, the levels of most cytokines, chemokines, and growth factors were increased in the plasma (Figure 10). It is to be noted that the primary tumor samples showed lower levels of cytokines, chemokines, and growth factors following both the treatment schedules. Distribution of CD45+CD11b+F4/80+CD206+ cells in the lungs, tumors and spleens was also analyzed in the animals that were treated for 2 and 4 weeks. The number of CD206+ cells is significantly lower (*p* < 0.005) in the lungs in the 2-week treatment group compared to that of the 4-week treatment group. On the other hand, the number of CD206+ cells is significantly higher (*p* < 0.013) in the tumors in the 2-week treatment groups compared to that of the 4-week treatment groups (Figure S8).

On the other hand, no significant differences were observed in the population of granulocytes, monocytes, and macrophages in the primary tumors or in the lungs in the 4T1-bearing animals. The number of cytotoxic T cells (CD45+CD3+CD8+) was significantly higher in the primary and metastatic TME in the engineered exosome-treated animals, and there was also an increased number of activated CD4+ T cells (CD45+CD3+CD25+CD69+) (Figure S9). We also observed an increased number of dendritic cells (DCs) in the lungs and spleen, although significant differences were not achieved (Figure S10). NK cells were present in the tumors, spleen, and peripheral blood.

### 3.7. Tumor Growth, Recurrence, Metastasis, and Survival

**Figure 11:**
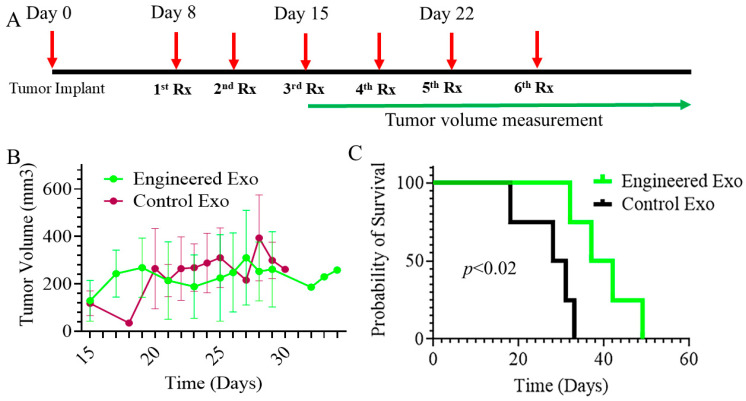
Primary tumor growth and survival: (**A**) Tumor implantation and treatment plans. (**B**) Tumor volume was measured at varying time points in animals bearing AT3 TNBC tumors (in syngeneic C57BL/6 mice) treated with the control and engineered exosomes. Six doses of the engineered exosomes were administered IV starting 8 days after tumor cell implantation. Data are expressed in mean ± SD. (**C**) Treatment of tumor-bearing animals with the engineered exosomes improved survival by depleting CD206+ M2 macrophages. Survival studies were analyzed by the Kaplan–Meier estimate and showed a significant difference (*p* < 0.02). *n* = 4 per group. Exo = exosomes, Rx = treatment.

The engineered exosome-treated animals showed relatively lower tumor volumes, although significant differences were not achieved. However, the engineered exosome-treated animals showed significantly longer survival compared to the control exosome-treated animals (Figure 11). Figure S11 shows the growth of individual tumors following exosome therapies.

**Figure 12:**
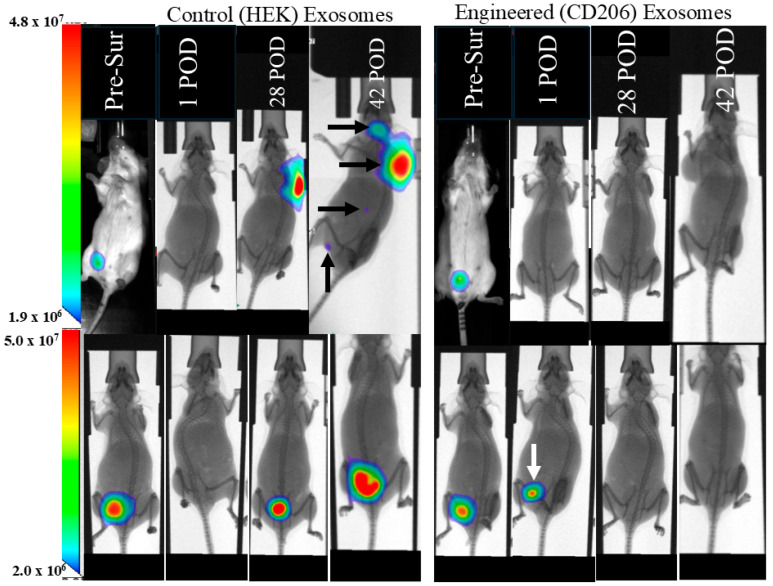
Resected model of breast cancer (4T1 in Balb/c mice) and metastasis: Representative images from tumor-resected animals (two from each group). All the primary tumors were resected 11 days after tumor cell implantation, and the animals were followed up to 63 days after resection. The figure shows representative animals from the control (HEK293 (**left panel**)) and engineered exosome-treated (**right panel**) groups. The animals treated with the control exosomes began showing local recurrence and lung metastasis (black arrows show local recurrence and multiple distal metastases) starting on day 28 post-resection. None of the animals that received the engineered exosomes to deplete CD206+ cells showed any recurrence or distal metastasis. It is noteworthy that a small leftover tumor (white arrow) in one of the engineered exosome-treated animals disappeared following therapy on subsequent bioluminescent imaging. *n* = 5 per group. Pre-Sur = pre surgery, POD = post-operative day.

**Figure 13:**
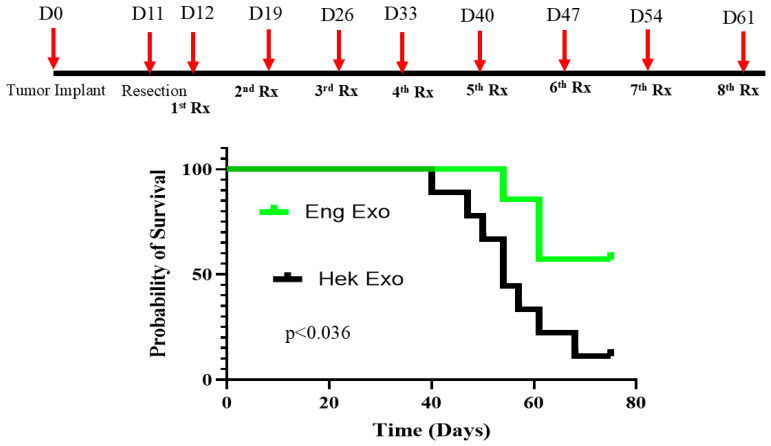
Survival of resected-tumor (AT3 in C57BL/6 mice)) animals: Primary tumors (AT3 in C57BL/6 mice) were resected on day 11 post-implant. The animals then received 8 doses (1 dose per week) of the HEK293 control or engineered exosomes starting on day one after the resection. Weekly BLI was performed, and the well-being of the animals was followed until the euthanasia criteria were fulfilled or at day 75. Treatment of the animals with the engineered exosomes had improved survival. Survival studies were analyzed by the Kaplan–Meier estimate and showed a significant difference (*p* < 0.036). *n* = 7–9 per group. Eng Exo = engineered exosomes, HEK = HEK293 exosomes, D = day, Rx = treatment.

In our previous studies, we have demonstrated the timeline (6–7 weeks) of the development of distal metastasis in the 4T1-resected primary tumor models [40]. The animals treated with the control exosomes began to show local recurrence and lung metastasis starting on day 28 post-resection. On the other hand, none of the animals that received the engineered exosomes to deplete CD206+ M2 macrophages showed any recurrence or distal metastasis. It is noteworthy that a small leftover tumor (white arrow) in one of the engineered exosome-treated animals disappeared following therapy on subsequent BLI (Figure 12). Figure S3 shows the serial BLI of the animals treated with HEK293 and engineered exosomes after resection of the primary tumor. The survival study using the AT3-resected model shows significantly enhanced survival when the animals were treated with the engineered exosomes (Figure 13).

## 4. Discussion

We successfully generated M2 macrophage-targeting engineered exosomes that carried CD206-targeting peptides and therapeutic payload (Fc-mIgG2b). We confirmed the presence of our inserted peptides or protein in the exosomes and showed their specificity in vitro and in vivo. Longterm administration of these exosomes did not show any sign of long-term effects in the lungs, heart, liver, spleen, or kidneys of the treated mice. These engineered exosomes depleted targeted CD206+ M2 macrophages and delayed the growth of metastatic foci in both models of syngeneic breast cancers.

Recent reports have indicated the importance of macrophages in breast cancer, and macrophages have become a key player in breast cancer therapy resistance [50,51]. We previously demonstrated the effect of engineered exosomes targeting CD206+ macrophages in animal models of breast cancer (4T1 tumor-bearing) [37]. The anti-macrophage agents that are currently being used in preclinical or early clinical trials target both M1 and M2 populations, which causes unwanted depletion of M1 macrophages. M1 macrophages are important for antigen presentation and cytotoxic T-cell-mediated tumor cell killing [52,53]. Antibody targeting tumor-associated macrophages (TAMs) (both M1 and M2) is in early stages of development [54,55]. Very recently, Jaynes et al. have shown the effectiveness of a small peptide (RP-182)-based therapeutics that activates the mannose receptor CD206 expressed on M2 macrophages to elicit endocytosis, phagosome–lysosome formation, autophagy, and reprogramming of M2-like TAMs to an antitumor M1-phenotype [56]. However, it is unclear from the manuscript whether the investigators have used any delivery vehicle for intraperitoneal injections. It is known that many hydrophobic peptide-based therapeutics or imaging agents are not water-soluble and require a carrier (usually nanoparticles) for IV injection [57]. In this study, we used recombinant DNA technology to make engineered exosomes that are biocompatible, biodegradable, and water-soluble for IV or IP administration. We demonstrated the effectiveness of the engineered exosomes in controlling recurrent tumor growth and enhancing survival. In our studies both AT3 and 4T1 bearing animals showed improved survival and delayed development of recurrent tumors and distal metastasis (resection models). In our studies we used immunologically distinct animal models [58] with a limited number of samples. Future studies are needed to confirm the findings using a larger sample size.

It is necessary to precisely target immunosuppressive M2 macrophages in the TME. It is noted that CD206+ macrophages are seen in a limited number in the lungs, spleen, and liver as resident macrophages [15]. Dendritic cells may also express mannose receptor/s under inflammatory skin disease conditions [59,60]. A subpopulation of hepatic endothelial cells in the liver may express the CD206 marker. However, our studies showed no changes in the liver and other major organs on hematoxylin–eosin (H&E) staining following 6 weeks of treatment with the engineered exosomes, and the flow cytometry did not show any depletion of dendritic cells. Our SPECT study also did not show increased uptake or retention of the engineered exosomes in the lungs or liver compared to that of the control exosomes, indicating that the engineered exosomes should not have detrimental effects in these organs. Based on the above discussion and published reports of different agents targeting TAMs in tumors [37,54,55,56], our strategies of using engineered exosomes derived from HEK293 cells in targeting immunosuppressive M2 macrophages in the TME have great translational potential. Notably, HEK293-derived exosomes have shown no immune activation or suppression following long-term injections in animal models [61]. Consistent with these findings, we did not observe changes in cytokine profiles or organ morphology after multiple injections of HEK293-derived exosomes. These exosomes contained cell-targeting moieties and Fc-mIgG2b, further supporting their apparent immunological safety.

The engineered exosomes targeting CD206+ M2 macrophages showed comparable specificity to that of targeting peptides [37], and the exosomes are stable at −20 °C or at −80 °C for a longer shelf life, and can be produced continuously using stably transduced HEK293 cells. Our long-term-stored (frozen) engineered or control exosomes showed comparable size distribution, similar morphology (by TEM), and retained the inserted protein (Fc-mIgG2b by Western blotting). Compared with synthetic nanoparticles like liposomes or metallic particles, exosomes offer advantages such as better biocompatibility, low toxicity, minimal immunogenicity, enhanced permeability (including through the BBB), and stability in biological fluids. Additionally, exosomes accumulate more specifically in target cells without being trapped in endosome–lysosome pathways [24,25,26,27,28,29,62]. As the exosome can cross the blood–brain barrier easily, investigators have used engineered exosomes targeting brain cancer and stroke [63,64,65,66]. Our engineered exosomes could have the potential for clinical translation.

ADCC is a non-phagocytic mechanism by which most NK cells (effector cells) can kill antibody-coated target cells in the absence of complement and without the major histocompatibility complex (MHC) [67,68,69,70]. Antibodies serve as a bridge between Fc receptors on effector cells and the target antigen on the cells to be killed. ADCC occurs through various pathways, including (a) release of cytotoxic granules; (b) TNF family death receptors signaling; and (c) release of pro-inflammatory cytokines, such as IFN-γ [71]. Here, we utilized the Fc gamma-receptor (FcγR)-based platform to deplete M2 macrophages, using the sequence of Fc-mIgG2b that triggers FcγR-mediated phagocytosis and cytotoxicity [37,72]. This study has shown an abundant number of NK cells in the tumors, spleens, and peripheral blood of the tumor-bearing animals, indicating that an engineered exosome-based approach for ADCC is valid and can be translational.

Previous investigations have proved the enhanced accumulation of systemically administered exosomes at the sites of lesions [36,62,73]. Due to their cellular origin, exosomes exhibit enhanced permeability, which is an advantage over synthetic nanoparticles [74,75,76,77]. Exosomes have also been shown to utilize enhanced permeability and retention (EPR) effects [77,78]. Due to their higher stability in biological fluids and enhanced permeability, exosomes are better for the targeted delivery of therapeutic payloads [74,75,76,77,79]. Our current studies showed active accumulation of engineered exosomes at the sites of primary and metastatic breast cancer foci and depletion of CD206+ macrophages in the spleens. This depletion of CD206+ cells was related to NK cell-mediated ADCC; additionally, there was increased activity of T-cells in the primary tumors and metastatic sites (lungs).

There is always a risk that depleting immunosuppressive cells may initiate hyperactive inflammation or immunogenic reactions. We have studied the effect of depletion of immunosuppressive CD206+ macrophages in normal and tumor-bearing animals. We have not observed any changes in the levels of inflammation, immunogenicity, immunosuppressive cytokines, or other growth factors in the plasma in two different syngeneic immunocompetent animals (C57BL/6 and Balb/c mice) following six doses of therapeutic and control exosomes. However, we have noticed differential expression of different cytokines in metastatic sites (lungs) and plasma at 2 and 4 weeks, respectively, following administration of therapeutic engineered exosomes either intravenously or intratumorally compared to that of targeting engineered exosomes (engineered exosomes without therapeutic payload). We observed more inflammatory cytokines in the metastatic sites (lung tissues) with a significantly lower number of CD206+ M2 macrophages after 2 weeks of treatments with engineered exosomes, indicating active depletion of immunosuppressive macrophages and accumulation of cytotoxic T-cells (both flow cytometry and membrane array). These findings support the idea that depletion of immunosuppressive macrophages will change the TME to a more immunogenic status and may open the window for immunotherapy using immune checkpoint inhibitors [80,81,82]. However, the changes in the cytokine level at the metastatic site were not sustained when the treatments continued for 4 weeks, but there was a global increase in cytokine and growth factor levels in the plasma with a significantly lower number of CD206+ M2 macrophages in the primary tumors. These combined findings are difficult to explain, but the global increase in cytokine and growth factor levels in the plasma at 4 weeks could be an indication of the development of therapy resistance to initiate distal metastasis. Many investigators have already pointed out the development of therapy resistance following myeloid cells or macrophage-targeted therapies in different tumor models due to the heterogeneity of macrophages and the dynamic nature of the TME that leads to treatment failure [83]. Therefore, a newer approach is needed to counteract the effect of immunosuppressive M2 macrophages.

## 5. Conclusions

In this study, we have used two different treatment models: (1) treatment was given to breast cancer-bearing animals starting on day 8 following implantation and continued for 2 weeks (6 doses) to mimic the neoadjuvant model, and (2) treatment was given to animals following resection of implanted tumors on day 11 following implantation to mimic the post-surgical model. In both cases, engineered exosomes targeting M2 macrophages improved the survival and delayed the growth of metastatic foci, indicating the effectiveness of the developed engineered exosomes in altering the TME and preventing the invasive and metastatic potential of breast cancer cells. M2 macrophages are known to induce immune suppression and inhibit inflammation, epithelial-to-mesenchymal transition, invasion, angiogenesis, and subsequent tumor progression, particularly in TNBC [43,84,85,86].

## 6. Potential Clinical Applications, Benefits, and Risks

Exosomes are natural, nano-sized vesicles (30–150 nm) involved in intercellular communication. They can be engineered to carry specific proteins for targeted therapy. We have generated engineered exosomes from FDA-approved HEK293 cells, which are widely used to produce therapeutic proteins and gene therapy vectors. Engineered exosomes developed in HEK293 cells can easily be translated to the clinic, provided the optimum dose is determined by dose escalation studies and toxicity studies are thoroughly carried out. The usefulness of M2 macrophage-targeting engineered exosomes can be determined in conjunction with immunotherapy. Clinical trials using engineered exosomes (e.g., mesenchymal stem cell-derived exosomes carrying siRNA for pancreatic cancer) are already underway (iEXPLORE Trial (NCT03608631)). Our approach holds significant promise for rapid clinical translation to modulate immunosuppressive TME and improve survival in patients with metastatic breast cancer.

## 7. Study Limitations

There are many limitations in our study. The number of animals per group was limited; however, both flow cytometry analysis and survival studies showed the effectiveness of the engineered exosomes in depleting M2 macrophages and improving survival significantly, respectively. Although the engineered exosomes inhibited recurrence/metastasis following excision of the primary tumor and improved the survival of both groups of mice, the engineered exosomes did not inhibit the growth of the primary tumors significantly. This could be due to the low number of animals in the primary tumor groups. The experiments were performed across independent batches of exosomes with consistent trends. We did not collect lymph nodes for the analysis of toxicity and cellular composition. Instead, we used the spleen as an example of lymphoid organs. The rationale behind using the spleen was to show the effects on the resident macrophages that are more pronounced and located in various areas of the spleen, which can be easily determined [87]. We also did not collect bone marrow to determine its cellular compositions, as our previous study indicated only modest changes in bone marrow following breast cancer implantation and therapy [45].

Our studies were set to be a proof-of-principle study, and we have not determined the mechanisms by which engineered exosomes made bridges between NK cells and M2 macrophages in the TME or lungs, or how ADCC was initiated. Mechanistic studies can be performed in our future investigations. We did not include immunotherapy along with targeting M2 macrophages, which could be included in future investigations. Our study showed activated T cells and decreased CD206+ M2 macrophages in the metastatic sites during 2 weeks of treatment, which could be a better window to initiate immunotherapy.

## Data Availability

Most of the data is included in the manuscript. All the data will be available from the corresponding author upon demand.

## Supplementary Materials

The following supporting information can be downloaded at https://www.mdpi.com/article/10.3390/cancers18101619/s1, Figure S1: Graphical representation of vector design and mode of action of engineered exosome. (A) The graphical presentation shows the protocol to insert CD206+ M2 macrophage-targeting peptides (9aa) and payload (Fc-mIgG2b) between the signaling peptide of Lamp2b and its C-terminus. His-tag was used as reporter for the expression of inserted peptides/protein. mCherry was used as reporter for the successful transduction/selection of HEK293 cells using lentivectors. It is noteworthy that mCherry was not expressed in the exosomes. (B) The cartoon depicts modes of action of engineered exosome which functions as a bridge between effector cells (NK cells) and CD206+ M2 macrophages. Once NK cell and macrophage are bridged by engineered exosome, NK cells will release granzyme and perforin and cause the death of M2 macrophage by Apoptosis. CS = C-terminus segments, SS = signaling peptide segment, TP = targeting peptides; Figure S2: Integrity of frozen exosome stored for 18 to 24 months at −80 °C. (A) NTA analysis shows the distribution of exosome size. (B,C) TEM images show the size distribution and typical cupping of the exosome from engineered and HEK293 exosomes, respectively. (D) Western blotting shows the presence of inserted mIgG2b only in the engineered exosomes (18- to 24-months-old exosomes). All exosomes (including 18 months old HEK293 exosomes) show CD9. NTA = Nanoparticle tracking analysis, TEM = Transmission electron microscope, WB = Western blotting; Figure S3: (A) Pre surgery and (B) post-surgery BLI images show the presence of tumors in 4T1 tumor bearing animals (Balb/c mice). Note that one animal died after surgery and two animals had large residual tumors and were discarded. BLI images show the growth of 4T1 resected tumors in animals treated with HEK293 (C–E) and engineered (F–H) exosomes. POD = post-operative day; Figure S4: Difference between 9aa and 6aa targeting peptide containing engineered exosomes. Mouse bone marrow-derived macrophages (MBMDMs) were collected and grown in the macrophage media for 3–4 days and MBMDMs were polarized to M2 macrophages using IL13 and IL-4. Then DiI tagged engineered exosomes were added to the culture with or without blocking peptides. (A) Mouse embryonic fibroblasts (MEFs) were used as negative control, which showed no uptake of engineered exosome. (B) Engineered exosomes carrying the 9aa targeting peptide showed robust uptake to the MBMDMs-polarized M2 macrophages, (C) which could be completely blocked by pre-incubation with corresponding blocking peptides (9aa). (D) Although uptake of engineered exosomes carrying 6aa targeting peptide was also robust in the MBMDMs-polarized M2 macrophages; (E) pre-incubation with blocking peptide (6aa) could not completely block the uptake to the cells. Yellow arrows shown in (B–E) indicate DiI tagged exosomes (red) in the MBMDMs. *n* = 3 per group. MEF = mouse embryonic fibroblast, MBMDM = mouse bone marrow derived macrophages; Figure S5: Multi-color flow-panels were used for detecting CD45+/CD11b+ cells and their subpopulations in the primary tumors, spleen, and lungs (Balb/c or C57BL/6 mice). Cells were labeled with flow antibodies (Biolegend, Sandiego, CA, USA) using standard procedures following blocking of Fc-receptors. These are the examples of CD45+ cells present in the primary tumors and their subpopulations following vehicle (PBS) and engineered exosomes (CD206-Exo) treatments. *n* =3 per group. Exo = exosomes, PBS = phosphate buffered saline; Figure S6: Multi-color flow-panels were used for detecting CD45+/CD3+ cells and their subpopulations in the primary tumors, spleens, and lungs (Balb/c or C57BL/6 mice). Cells were labeled with flow antibodies (Biolegend) using standard procedures. These are examples of CD45+ cells present in the spleen and their subpopulations following vehicle (PBS) and engineered exosomes (CD206-Exo) treatments. *n* =3 per group. Exo = exosomes, PBS = phosphate buffered saline; Figure S7. Both Balb/c and C57BL/6 (B6) animals were treated with 6 doses of either PBS or engineered exosomes over 6 weeks. 6 months after the last dose, animals were euthanized, and different organs were collected, fixed and sections were made for H&E staining to determine the effect of engineered exosomes at the cellular level due to presence of resident macrophages in the organs. No changes observed following the treatment with engineered exosomes. The images were obtained using 40× optics and nine images were stitched together. Yellow bar = 300 µm. B6 = C57BL/6, PBS = phosphate buffered saline; Figure S8: The distribution of CD45+CD11b+F4/80+CD206+ cells in the lungs, tumors and spleens in animals were analyzed that were treated for 2 and 4 weeks (AT3 tumors in C57BL/6 mice). The number of CD206+ cells was significantly lower (* *p* < 0.005) in lungs in 2-week treatment group compared to that of 4-week treatment group. On the other hand, number of CD206+ cells was significantly higher (# *p* < 0.013) in tumor in 2-week treatment group compared to that of four-week treatment group; Figure S9: Tumor (4T1 in Balb/c mice) bearing animals were treated with vehicle (PBS) or engineered exosomes (CD206). At the end of the treatments, animals were euthanized, primary tumors and lungs were collected for single cell suspension. Multi-color panel flow cytometry was conducted to determine the myeloid and T-cell populations. (A) No significant differences were observed in the population of granulocytes and M1 macrophages both in primary tumors and metastatic sites. (B) Significantly increased number of different Tcell populations was observed in the primary and metastatic site of 4T1 TNBC following treatment with PBS and engineered exosomes targeting CD206+ cells. Total 100,000 counts were acquired from each sample for flow analysis. One way ANOVA was applied. * *p* < 0.05. *n* = 3 per group; Figure S10: Tumor-bearing mice (4T1 in Balb/c mice) were treated with vehicle (PBS) and engineered exosomes (CD206). At the end of the treatment different organs were collected, and then single cell suspension was made, and flow cytometry was performed. (A) Number of dendritic cells was increased in the metastatic foci following treatment with engineered exosomes, however, significance was not achieved. (B) Presence of NK cells was observed in the primary tumors, spleens, and peripheral blood. Total 100,000 counts were acquired from each sample for flow analysis. One way ANOVA was applied. * *p* < 0.05 compared to PBS-treated animals. *n* = 3 per group. PBS = phosphate buffered saline, PBMC = peripheral blood mononuclear cells; Figure S11: AT3 cells were implanted in C57BL/6 female mice. Treatment was started on day 8 and continued for three weeks. Six doses of HEK293 (HEK Exo) and engineered (Eng Exo) exosomes were administered (2 doses per week); Original PCR gel for Figure 1 showing CD206 and IgG2b; Original WB gel for Figure 1 showing CD9 band at 25 kDa.
