# Supplementary material for: Therapeutically Engineering Exosomes to Target CD206+ M2 Macrophages to Prevent the Development of Primary Tumors and Distal Metastases in Breast Cancers
Source: Cancers (Basel). 2026 May 16;18(10):1619. doi: 10.3390/cancers18101619 (PMC13204471; doi:10.3390/cancers18101619)

Supplemental information:

Title: Therapeutically Engineering Exosomes to Target CD206+ M2-Macrophage to Prevent the Development of Primary Tumor and Distal Metastasis in Breast Cancers

Authors: Mahrima Parvin[1]*, Ahmet Alptekin[1]*, Sawaiz Kashif[1], Fowzia A Selina[1], Mst Anika Bushra1, Mohammad Syam[1], Mohammad H Rashid[2], Alicia Arnold[3], Yutao Liu[4], Santhakumar Manicassamy[1], Hasan Korkaya[5], Ali S. Arbab[1]

Supplemental Figure S1: Strategies for making engineered exosomes and modes of action.

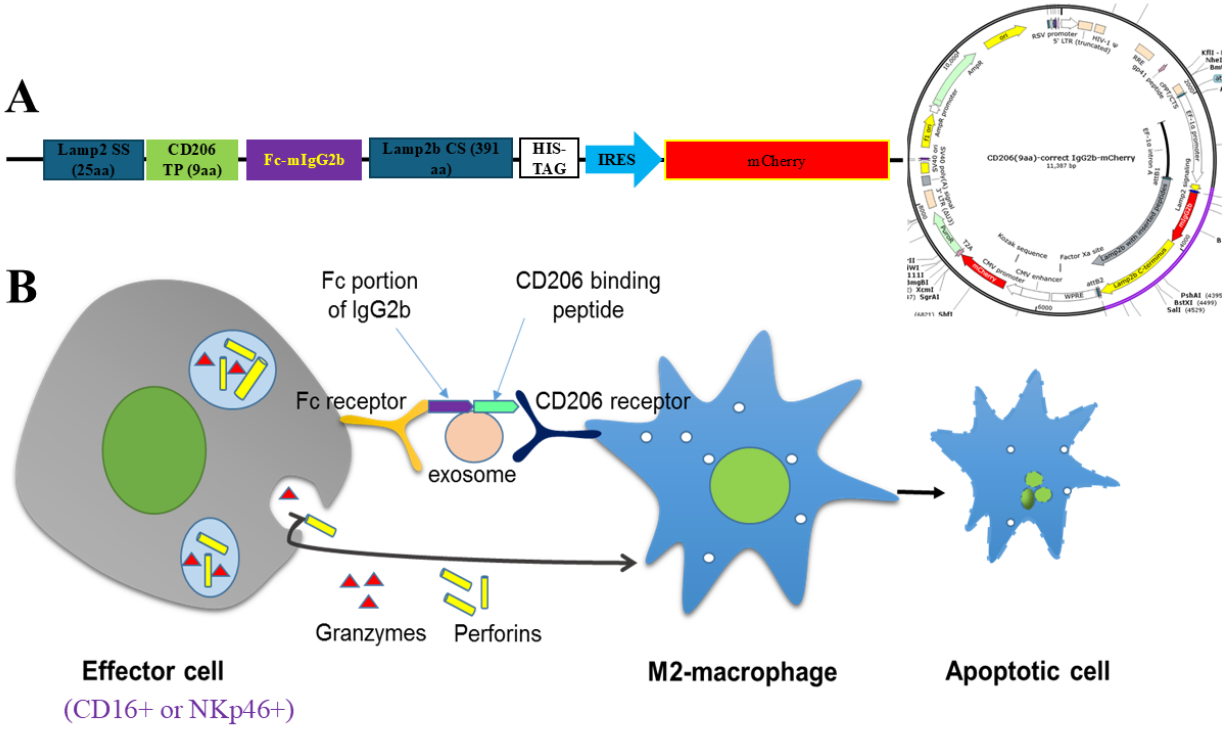

Figure S1: Graphical representation of vector design and mode of action of engineered exosome. (**A**) The graphical presentation shows the protocol to insert CD206+ M2 macrophage-targeting peptides (9aa) and payload (Fc-mIgG2b) between the signaling peptide of Lamp2b and its C-terminus. His-tag was used as reporter for the expression of inserted peptides/protein. mCherry was used as reporter for the successful transduction/selection of HEK293 cells using lentivectors. It is noteworthy that mCherry was not expressed in the exosomes. (**B**) The cartoon depicts modes of action of engineered exosome which functions as a bridge between effector cells (NK cells) and CD206+ M2 macrophages. Once NK cell and macrophage are bridged by engineered exosome, NK cells will release granzyme and perforin and cause the death of M2 macrophage by Apoptosis. *CS = C-terminus segments, SS = signaling peptide segment, TP = targeting peptides.*

Supplemental Figure S2: Integrity of frozen exosomes.

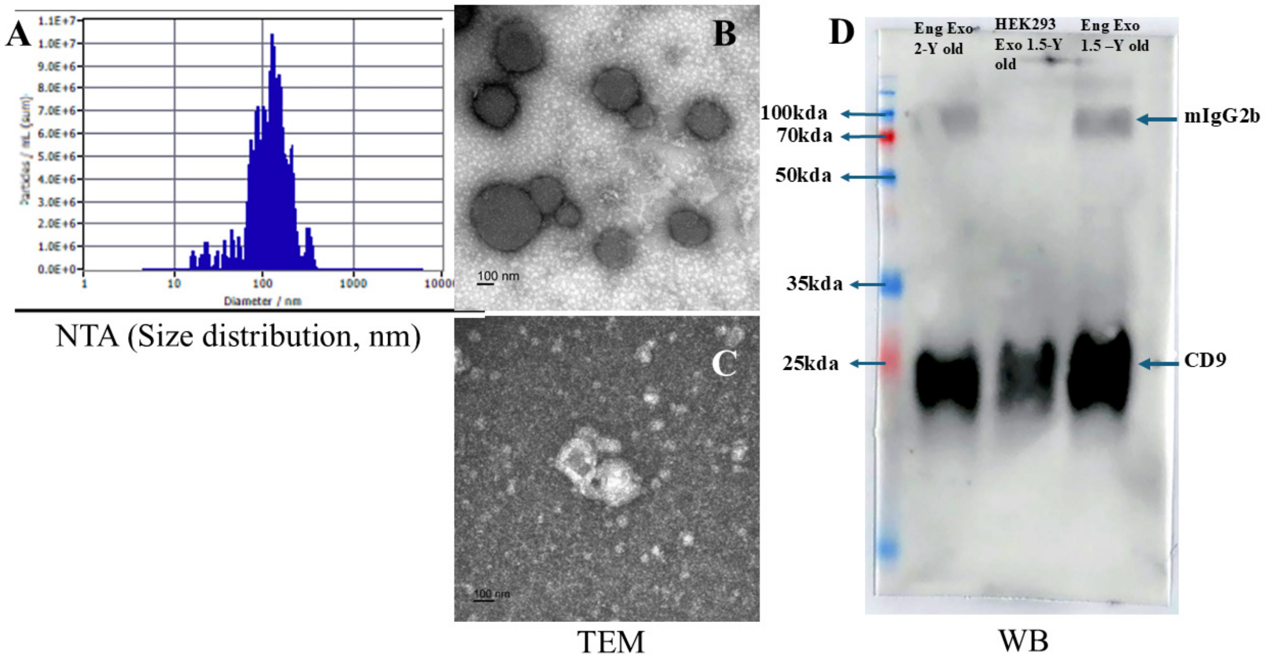

Figure S2: Integrity of frozen exosome stored for 18 to 24 months at −80°C. (A) NTA analysis shows the distribution of exosome size. (B,C) TEM images show the size distribution and typical cupping of the exosome from engineered and HEK293 exosomes, respectively. (D) Western blotting shows the presence of inserted mIgG2b only in the engineered exosomes (18- to 24-months-old exosomes). All exosomes (including 18 months old HEK293 exosomes) show CD9. *NTA = Nanoparticle tracking analysis, TEM = Transmission electron microscope, WB = Western blotting.*

Supplemental Figure S3: Development of recurrent and metastatic tumors in 4T1-resection model.

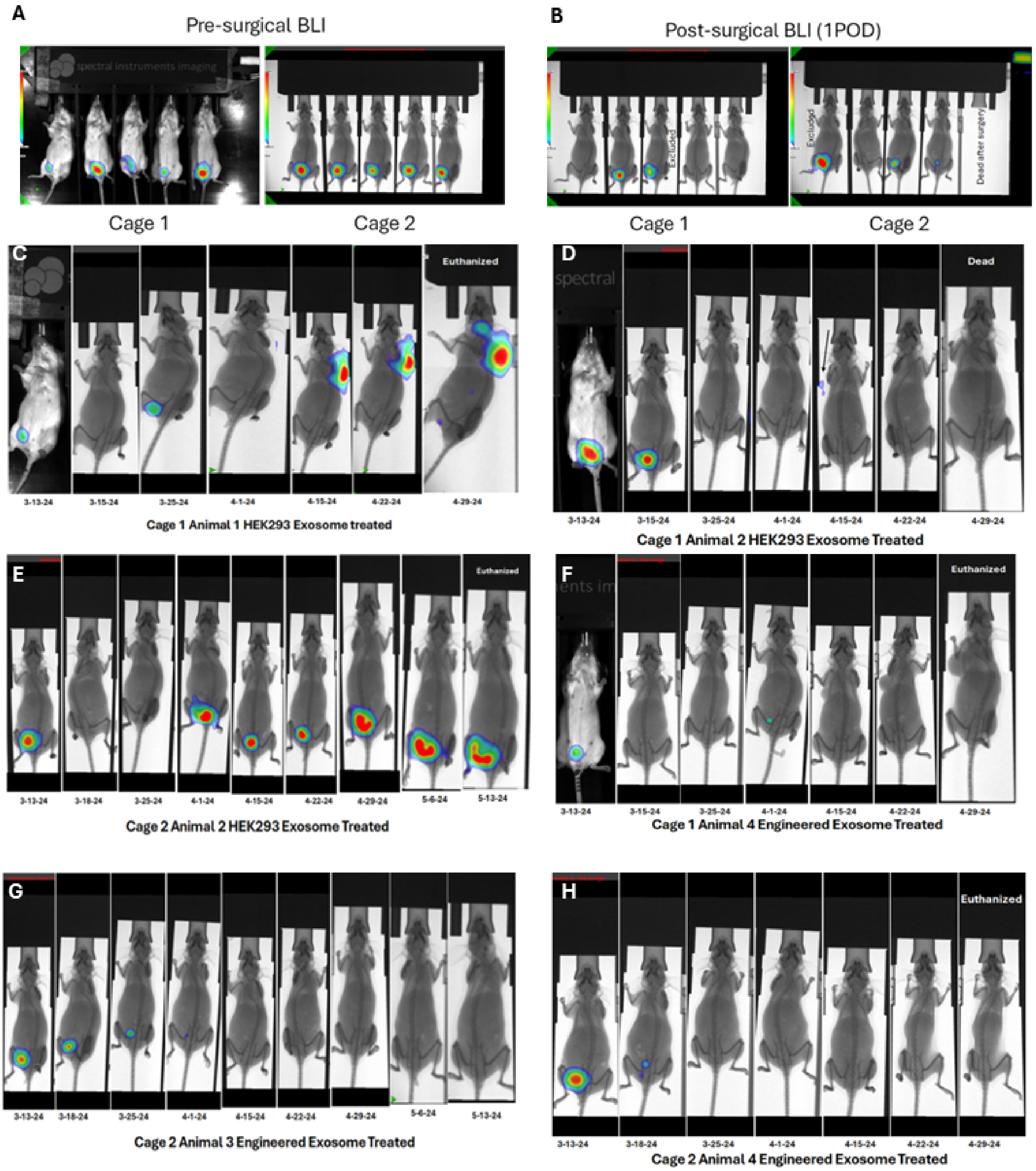

Figure S3: (A) Pre surgery and (B) post-surgery BLI images show the presence of tumors in 4T1 tumor bearing animals (Balb/c mice). Note that one animal died after surgery and two animals had large residual tumors and were discarded. BLI images show the growth of 4T1 resected tumors in animals treated with HEK293 (C–E) and engineered (F–H) exosomes. *POD = post-operative day.*

Supplemental Figure S4: Determination of specificity of 9aa peptide to target M2-macrophages.

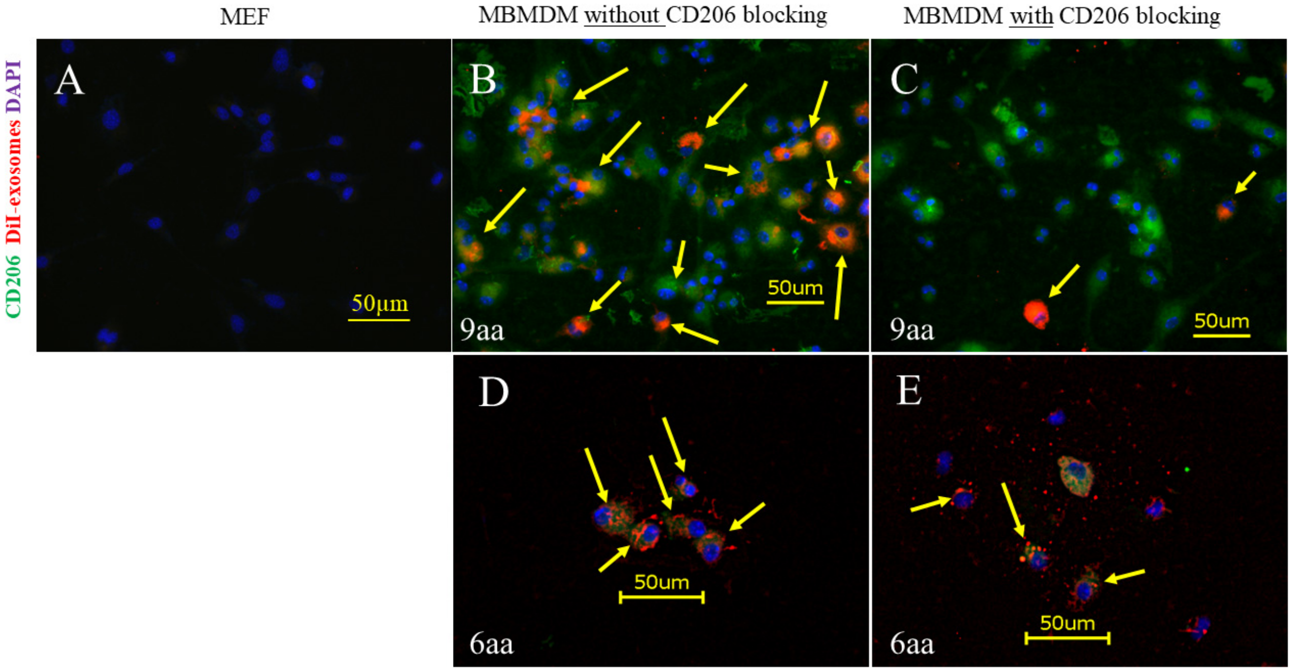

Figure S4: Difference between 9aa and 6aa targeting peptide containing engineered exosomes. Mouse bone marrow-derived macrophages (MBMDMs) were collected and grown in the macrophage media for 3–4 days and MBMDMs were polarized to M2 macrophages using IL13 and IL-4. Then DiI tagged engineered exosomes were added to the culture with or without blocking peptides. (**A**) Mouse embryonic fibroblasts (MEFs) were used as negative control, which showed no uptake of engineered exosome. (**B**) Engineered exosomes carrying the 9aa targeting peptide showed robust uptake to the MBMDMs-polarized M2 macrophages, (**C**) which could be completely blocked by pre-incubation with corresponding blocking peptides (9aa). (**D**) Although uptake of engineered exosomes carrying 6aa targeting peptide was also robust in the MBMDMs-polarized M2 macrophages; (**E**) pre-incubation with blocking peptide (6aa) could not completely block the uptake to the cells. Yellow arrows shown in (**B**–**E**) indicate DiI tagged exosomes (red) in the MBMDMs. *n* = 3 per group. *MEF = mouse embryonic fibroblast, MBMDM = mouse bone marrow derived macrophages.*

Supplemental Figure S5: Flow cytometry panel for myeloid cells.

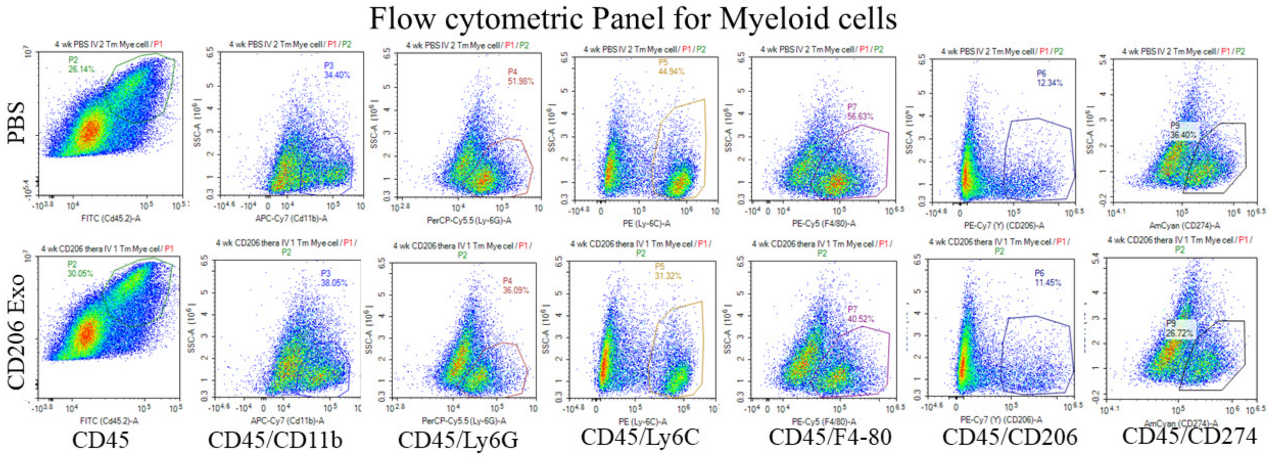

Figure S5: Multi-color flow-panels were used for detecting CD45+/CD11b+ cells and their subpopulations in the primary tumors, spleen, and lungs (Balb/c or C57BL/6 mice). Cells were labeled with flow antibodies (Biolegends) using standard procedures following blocking of Fc-receptors. These are the examples of CD45+ cells present in the primary tumors and their subpopulations following vehicle (PBS) and engineered exosomes (CD206-Exo) treatments. *n* =3 per group. *Exo = exosomes, PBS = phosphate buffered saline.*

Supplemental Figure S6: Flow cytometry panel for T-cells.

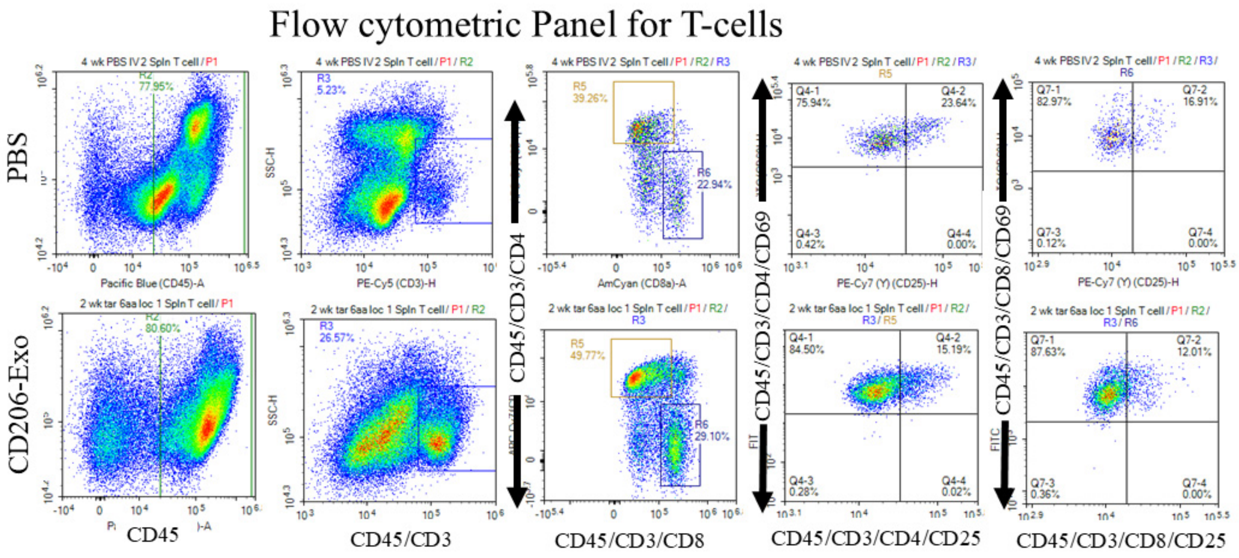

Figure S6: Multi-color flow-panels were used for detecting CD45+/CD3+ cells and their subpopulations in the primary tumors, spleens, and lungs (Balb/c or C57BL/6 mice). Cells were labeled with flow antibodies (Biolegends) using standard procedures. These are examples of CD45+ cells present in the spleen and their subpopulations following vehicle (PBS) and engineered exosomes (CD206-Exo) treatments. *n* =3 per group. *Exo = exosomes, PBS = phosphate buffered saline.*

Supplemental Figure S7: Hematoxylin and eosin (H&E) staining of different organs.

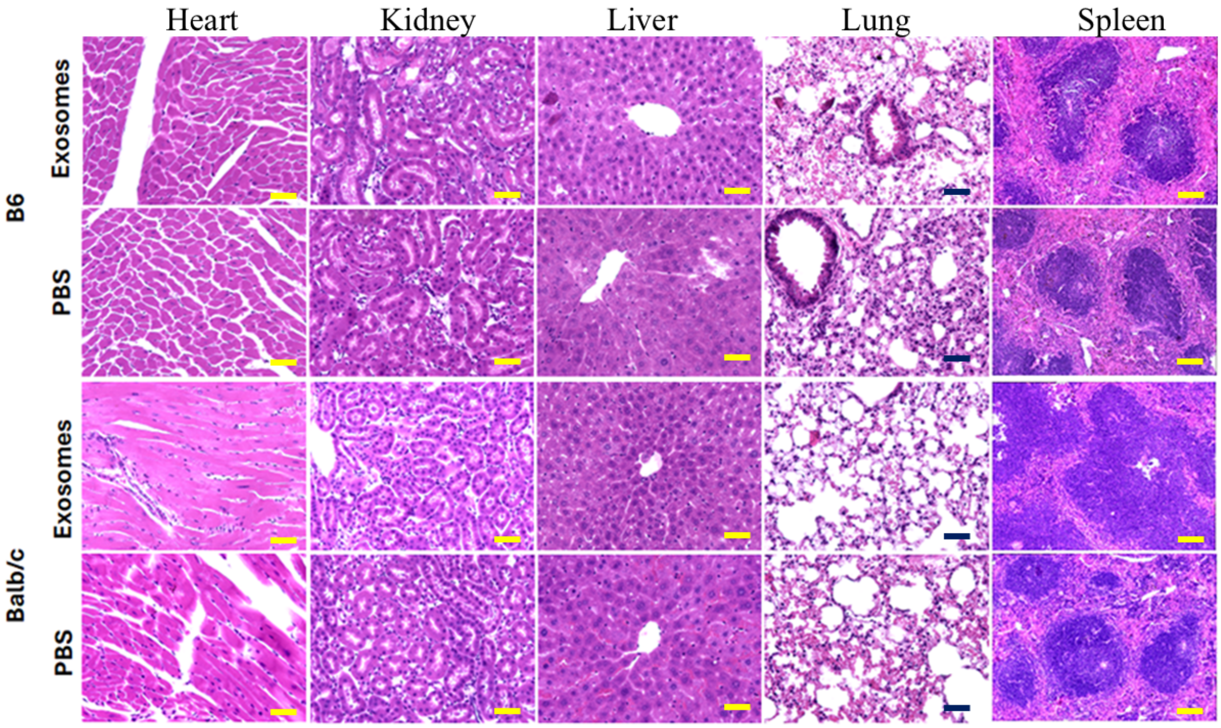

Figure S7. Both Balb/c and C57BL/6 (B6) animals were treated with 6 doses of either PBS or engineered exosomes over 6 weeks. 6 months after the last dose, animals were euthanized, and different organs were collected, fixed and sections were made for H&E staining to determine the effect of engineered exosomes at the cellular level due to presence of resident macrophages in the organs. No changes observed following the treatment with engineered exosomes. The images were obtained using 40× optics and nine images were stitched together. Yellow bar = 300 µm. *B6 = C57BL/6, PBS = phosphate buffered saline.*

Supplemental Figure S8: Distribution of CD45+CD11b+F4/80+CD206+ cells.

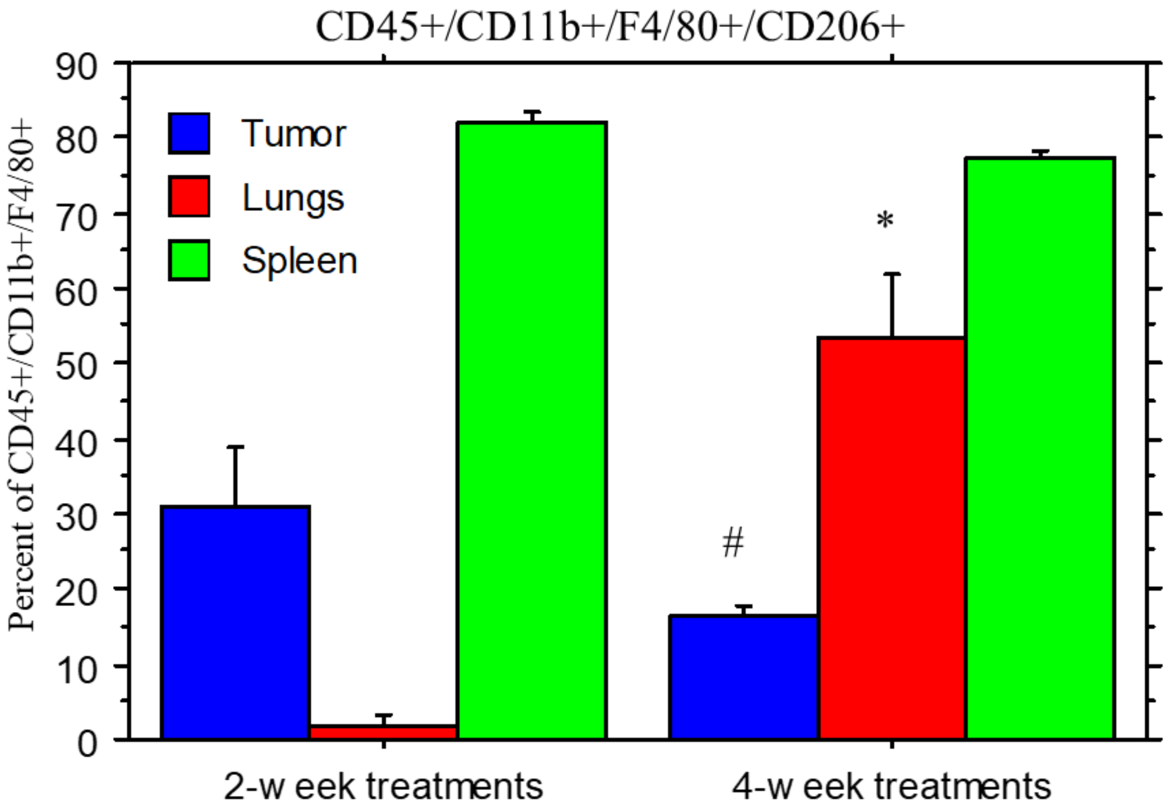

Figure S8: The distribution of CD45+CD11b+F4/80+CD206+ cells in the lungs, tumors and spleens in animals were analyzed that were treated for 2 and 4 weeks (AT3 tumors in C57BL/6 mice). The number of CD206+ cells was significantly lower (* $p < 0.005$) in lungs in 2-week treatment group compared to that of 4-week treatment group. On the other hand, number of CD206+ cells was significantly higher (# $p < 0.013$) in tumor in 2-week treatment group compared to that of four-week treatment group.

Supplemental Figure S9: Distribution of myeloid and T-cell population in the primary tumor and metastatic sites (lungs).

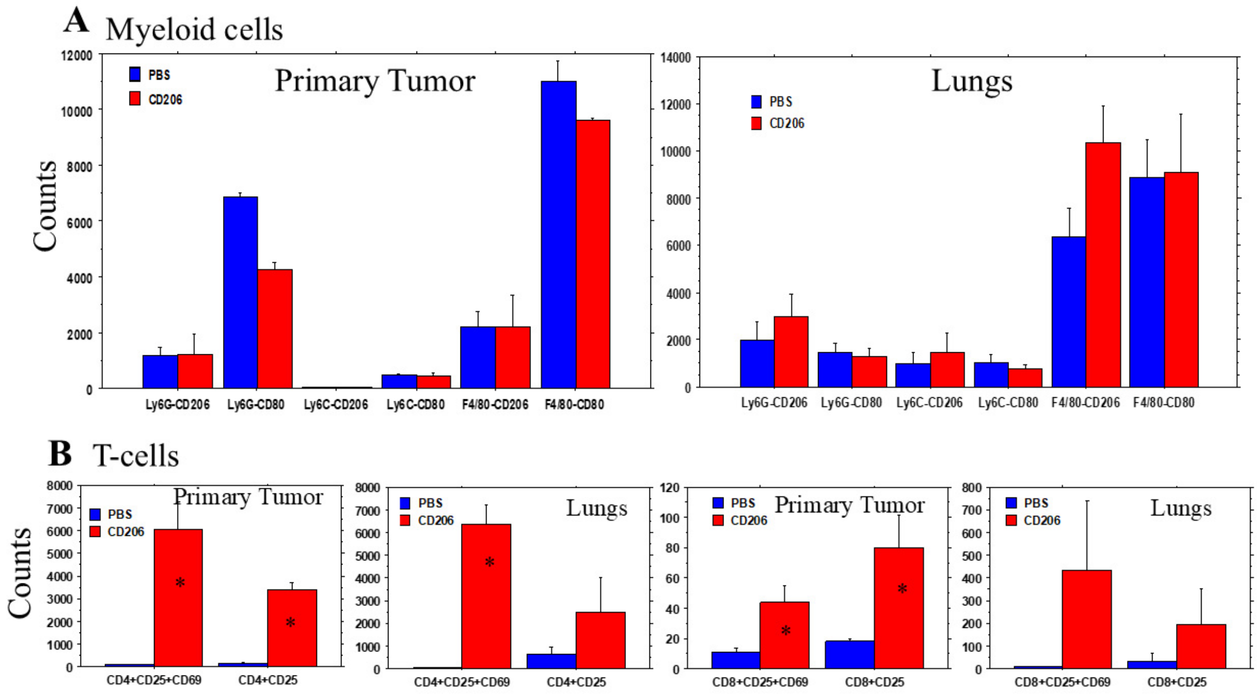

Figure S9. Tumor (4T1 in Balb/c mice) bearing animals were treated with vehicle (PBS) or engineered exosomes (CD206). At the end of the treatments, animals were euthanized, primary tumors and lungs were collected for single cell suspension. Multi-color panel flow cytometry was conducted to determine the myeloid and T-cell populations. (**A**) No significant differences were observed in the population of granulocytes and M1 macrophages both in primary tumors and metastatic sites. (**B**) Significantly increased number of different Tcell populations was observed in the primary and metastatic site of 4T1 TNBC following treatment with PBS and engineered exosomes targeting CD206+ cells. Total 100,000 counts were acquired from each sample for flow analysis. One way ANOVA was applied. * $p < 0.05$. $n = 3$ per group.

Supplemental Figure S10: Presence of dendritic and NK cells.

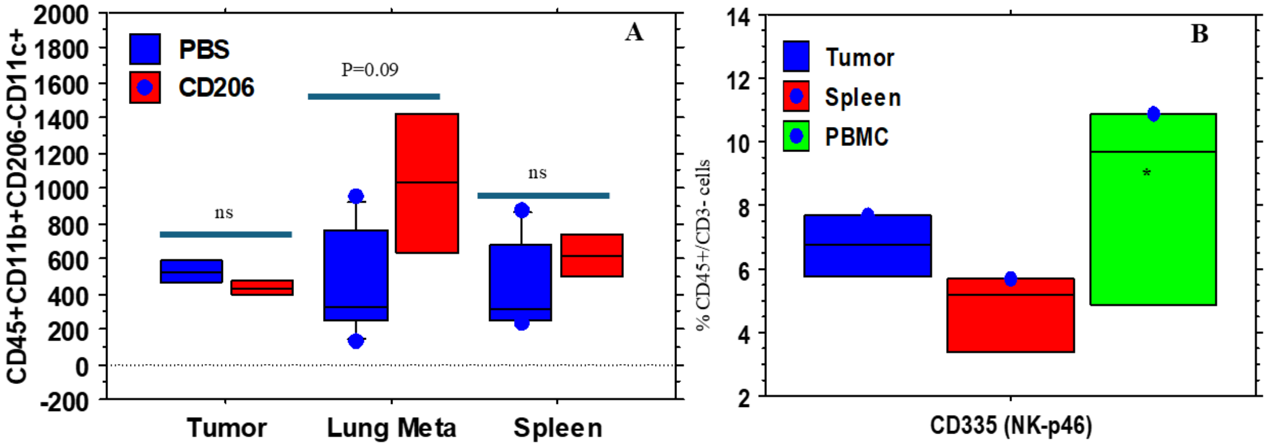

Figure S10. Tumor-bearing mice (4T1 in Balb/c mice) were treated with vehicle (PBS) and engineered exosomes (CD206). At the end of the treatment different organs were collected, and then single cell suspension was made, and flow cytometry was performed. (**A**) Number of dendritic cells was increased in the metastatic foci following treatment with engineered exosomes, however, significance was not achieved. (**B**) Presence of NK cells was observed in the primary tumors, spleens, and peripheral blood. Total 100,000 counts were acquired from each sample for flow analysis. One way ANOVA was applied. * $p<0.05$ compared to PBS-treated animals. $n = 3$ per group. PBS = phosphate buffered saline, PBMC = peripheral blood mononuclear cells.

Supplemental Figure S11: Development of individual tumors shown in Figure 11 in the main text.

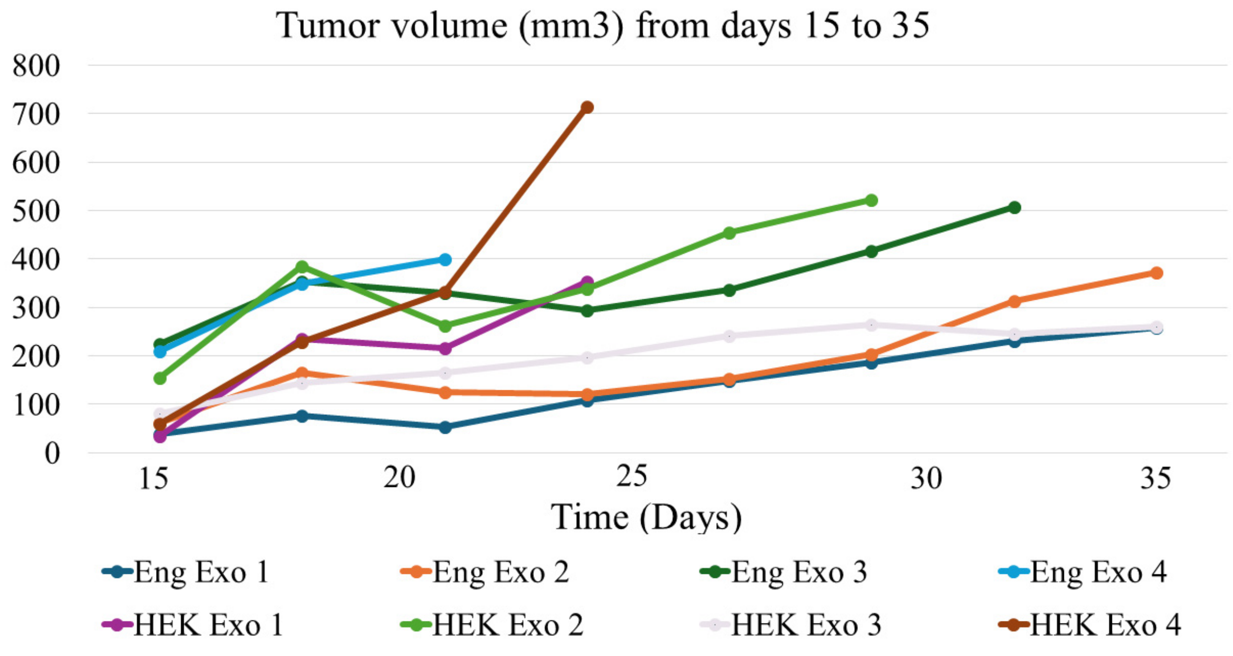

Figure S11. AT3 cells were implanted in C57BL/6 female mice. Treatment was started on day 8 and continued for three weeks. Six doses of HEK293 (HEK Exo) and engineered (Eng Exo) exosomes were administered (2 doses per week).

Original PCR gel for Figure 1 showing CD206 and IgG2b:

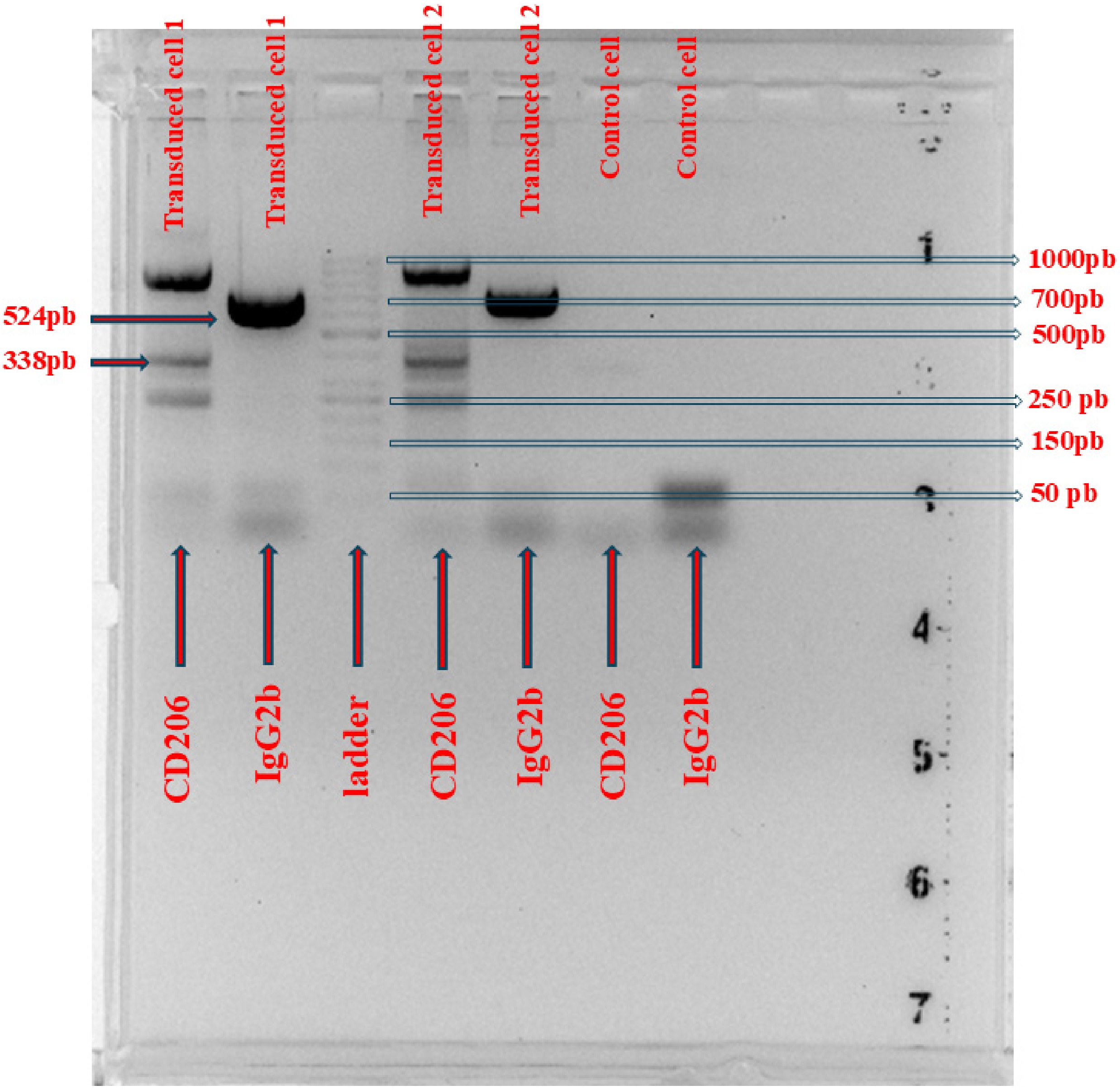

Original WB gel for Figure 1 showing CD9 band at 25 kDa:

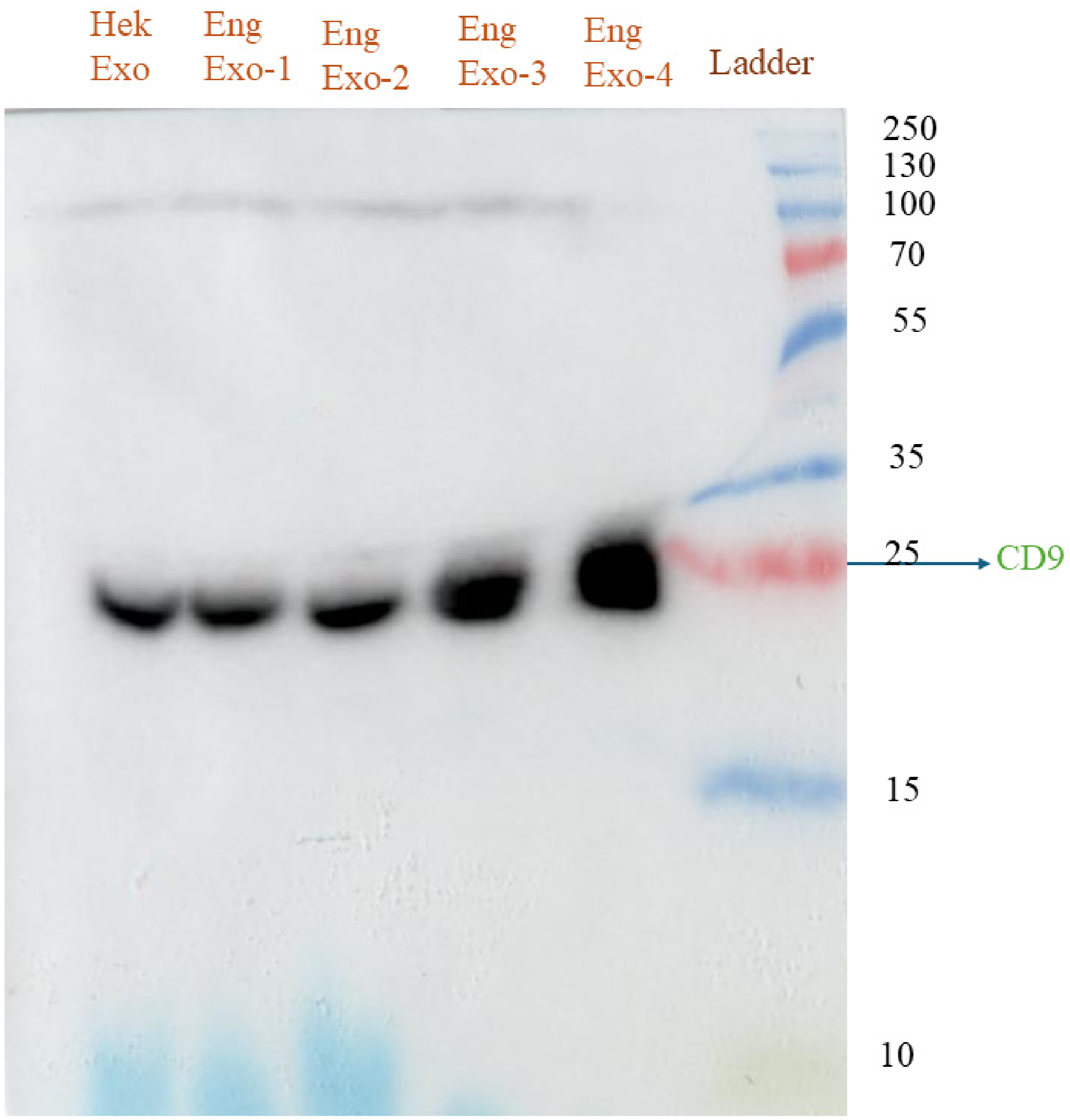

Supplement: Supplementary file 1 [file cancers-18-01619-s001.zip › cancers-4245966-supplementary.pdf]
